# GDF-5 can act as a context-dependent BMP-2 antagonist

**DOI:** 10.1186/s12915-015-0183-8

**Published:** 2015-09-18

**Authors:** Uwe Klammert, Thomas D. Mueller, Tina V. Hellmann, Kristian K. Wuerzler, Alexander Kotzsch, Anna Schliermann, Werner Schmitz, Alexander C. Kuebler, Walter Sebald, Joachim Nickel

**Affiliations:** Lehrstuhl für Mund-, Kiefer- und plastische Gesichtschirurgie, Universitätsklinikum Würzburg, Pleicherwall 2, 97070 Würzburg, Germany; Lehrstuhl für molekulare Pflanzenphysiologie und Biophysik, Julius-von- Sachs-Institut für Biowissenschaften, Universität Würzburg, Julius-von-Sachs- Platz 2, D-97082 Würzburg, Germany; Lehrstuhl für Biochemie und Molekularbiologie, Theodor-Boveri-Institut für Biowissenschaften, Universität Würzburg, Am Hubland, 97074 Würzburg, Germany; Lehrstuhl für Physiologische Chemie II, Theodor-Boveri-Institut für Biowissenschaften, Universität Würzburg, Am Hubland, 97074 Würzburg, Germany; Lehrstuhl für Tissue Engineering und Regenerative Medizin, Universitätsklinikum Würzburg, Röntgenring 11, D-97070 Würzburg, Germany; Fraunhofer-Institut für Grenzflächen- und Bioverfahrenstechnik IGB, Translationszentrum »Regenerative Therapien für Krebs- und Muskuloskelettale Erkrankungen« – Institutsteil Würzburg, Würzburg, Germany

**Keywords:** Antagonist, Crystal structure, Growth and differentiation factor 5, Ligand-receptor complex

## Abstract

**Background:**

Bone morphogenetic protein (BMP)-2 and growth and differentiation factor (GDF)-5 are two related transforming growth factor (TGF)-β family members with important functions in embryonic development and tissue homeostasis. BMP-2 is best known for its osteoinductive properties whereas GDF-5—as evident from its alternative name, cartilage derived morphogenetic protein 1—plays an important role in the formation of cartilage. In spite of these differences both factors signal by binding to the same subset of BMP receptors, raising the question how these different functionalities are generated. The largest difference in receptor binding is observed in the interaction with the type I receptor BMPR-IA. GDF-5, in contrast to BMP-2, shows preferential binding to the isoform BMPR-IB, which is abrogated by a single amino acid (A57R) substitution. The resulting variant, GDF-5 R57A, represents a “BMP-2 mimic” with respect to BMP receptor binding. In this study we thus wanted to analyze whether the two growth factors can induce distinct signals via an identically composed receptor.

**Results:**

Unexpectedly and dependent on the cellular context, GDF-5 R57A showed clear differences in its activity compared to BMP-2. In ATDC-5 cells, both ligands induced alkaline phosphatase (ALP) expression with similar potency. But in C2C12 cells, the BMP-2 mimic GDF-5 R57A (and also wild-type GDF-5) clearly antagonized BMP-2-mediated ALP expression, despite signaling in both cell lines occurring solely via BMPR-IA. The BMP-2- antagonizing properties of GDF-5 and GDF-5 R57A could also be observed in vivo when implanting BMP-2 and either one of the two GDF-5 ligands simultaneously at heterotopic sites.

**Conclusions:**

Although comparison of the crystal structures of the GDF-5 R57A:BMPR-IA_EC_- and BMP-2:BMPR-IA_EC_ complex revealed small ligand-specific differences, these cannot account for the different signaling characteristics because the complexes seem identical in both differently reacting cell lines. We thus predict an additional component, most likely a not yet identified GDF-5-specific co-receptor, which alters the output of the signaling complexes. Hence the presence or absence of this component then switches GDF-5′s signaling capabilities to act either similar to BMP-2 or as a BMP-2 antagonist. These findings might shed new light on the role of GDF-5, e.g., in cartilage maintenance and/or limb development in that it might act as an inhibitor of signaling events initiated by other BMPs.

**Electronic supplementary material:**

The online version of this article (doi:10.1186/s12915-015-0183-8) contains supplementary material, which is available to authorized users.

## Background

Functional synovial joints are essential to ensure proper biomechanical function of the skeleton and are formed from a mesenchymal interzone emerging at each prospective joint site [1, 2]. Despite the understanding that this interzone region is essential for joint formation, the specific developmental roles, fate, and mechanisms of action of the participating cells remain poorly understood [3]. Molecular and genetic studies have revealed that interzone cells express a number of genes from the Wnt and transforming growth factor (TGF)-β pathway including *wnt-9a*, *wnt-4*, *noggin*, and *gdf-5* [4–7]. Most importantly, proper joint formation requires the intervention of gene products with both chondrogenic and anti-chondrogenic activities. Aside from growth and differentiation factor (GDF)-5, several other members of the bone morphogenetic protein (BMP) family are also expressed in stripes at sites of joint formation, including those encoded by the genes *gdf-6*, *bmp-2*, and *bmp-4* [8–11]. Several mutations in either *gdf-5* or the closely related *gdf-6* gene impair joint formation at specific locations, thereby strongly indicating that these molecules are absolutely essential for the joint formation process [6, 9].

GDF-5, like BMP-2 and BMP-4, binds to and oligomerizes two types of serine-/threonine kinase transmembrane receptors, termed TGF-β superfamily type I and type II receptors [12, 13]. Of the seven different known type I receptors, BMPR-IA and BMPR-IB have been implicated in skeletal patterning. Mice with null mutations in the *bmpr-1b* gene are viable, but show defects in bone and joint formation closely resembling those seen in mice lacking GDF-5 [10, 14]. In contrast, mice with null mutations in *bmpr-1a* die early during embryogenesis [15]. However, a conditional knockout of *bmpr-1a* under the control of a GDF5-Cre driver allows the bypassing of embryonic lethality and yields viable mice with seemingly normal joints. But the progressive loss of articular cartilage within the joints of those conditional knockout mice strongly resembles the disease mechanism of osteoarthritis [16]. This observation clearly highlights the importance of BMPR-IA in cartilage homeostasis and repair.

In vitro interaction analysis demonstrated that GDF-5 binds the extracellular domains of the individual type II receptors ActR-II, ActR-IIB, and BMPR-II as well as the type I receptor BMPR-IB, with similar binding affinities as found for BMP-2 [17]. The most striking difference has been detected in the binding properties of BMP-2 and GDF-5 to the type I receptor BMPR-IA. Whereas BMP-2 binds to both type I receptors—BMPR-IA and BMPR-IB—with similar affinities, GDF-5 exhibits a 12-fold higher affinity for BMPR-IB than for BMPR-IA. This type I receptor specificity of GDF-5 is due to a single amino acid, Arg57, which is located in the pre-helix loop of the ligand [18, 19]. Mutation of Arg57 in GDF-5 to alanine abrogates type I receptor specificity completely, whereas the mutation of Arg57 to leucine leads to enhanced binding to BMPR-IA and attenuated type I receptor specificity [18, 19]. The latter mutation is found in patients with proximal symphalangism (SYM1), a rare disease characterized by a joint fusion of the middle and distal phalanges of the fourth digit. Its phenotype is very similar to that caused by mutations in Noggin, a modulator protein counteracting GDF-5 activity [18], and rather different to other mutations in GDF-5 found in patients with brachydactyly and Du Pan syndrome (also known as fibula aplasia complex brachydactyly). Mutations leading to the latter two phenotypes usually disrupt GDF-5 receptor binding and/or activity [18, 20] and are characterized by hypoplasia or aplasia of individual middle phalanges of different digits. Because only the binding affinity for BMPR-IA is increased in both GDF-5 variants (R57A and R57L), it can be assumed that the resulting SYM1 phenotype is due to a shift in receptor specificity. That GDF-5 can indeed signal via BMPR-IA is evident from the observation that BMP-2 and the GDF-5 variant R57L induce expression of alkaline phosphatase (ALP) in the chondrocyte cell line ATDC-5 with similar efficiency [18], a cell line lacking BMPR-IB [21]. Consistently, wild-type GDF-5 induces ALP expression in these cells but, compared to GDF-5 R57L and GDF-5 R57A, a 10-fold higher concentration is required for a half maximal response (EC_50_) [18, 19], which nicely correlates with the different binding affinities of these different GDF-5 proteins for BMPR-IA. Surprisingly and in contrast to the above observation, in C2C12 cells, a promyoblast cell line also carrying the BMPR receptor BMPR-IA, ALP expression can be induced by BMP-2, but not by GDF-5 [21]. Furthermore, the GDF-5 variant R57L, despite being a BMP-2 mimic with a similar BMPR-IA binding profile as BMP-2, stimulates only weak ALP expression [18], indicating that the ALP expression by GDF-5 is cell-line specific.

To further investigate this discrepancy in signaling outcome, we examined the biological activities of BMP-2, GDF-5, and GDF-5 R57A in vitro using cell-based assays. We compared the results with data from in vivo models, in which ligand-doped carriers were implanted into rat hind limbs (heterotopic model) or calvaria (orthotopic model). Structural studies were performed by crystallizing the binary complex of GDF-5 R57A bound to BMPR-IA and determining its structure at high resolution. On the basis of this structure the molecular mechanism by which GDF-5 possibly discriminates between the two type I receptors BMPR-IA and BMPR-IB could be analyzed. The biological activities of GDF-5 analyzed in cell-based assays as well as in vivo could then be correlated to aspects of ligand receptor assembly.

## Results

### Structure of the binary complex of GDF-5 R57A bound to BMPR-IA

**Fig. 1:**
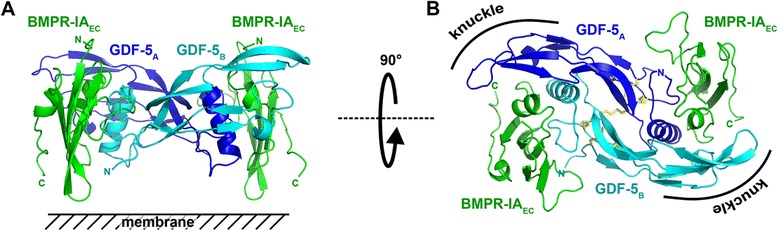
Architecture of the binary GDF-5 R57A:BMPR-IA_EC_ complex. Ribbon plot of the binary complex of GDF-5 R57A (monomer subunits colored in *blue* and *cyan*) bound to the extracellular domain of BMPR-IA (*green*). **a** View from the side, the side facing the putative cell surface is indicated. **b** As in (**a**) but viewed from the top showing the two-fold symmetry of the homodimeric GDF-5 as well as of the ligand-receptor complex. Disulfide bonds forming the two cystin-knot motifs in the GDF-5 monomer subunits and the intermolecular disulfide bond are shown as *yellow sticks*. The location of the knuckle epitope important for BMP type II receptor binding is indicated

Similar to other BMP-BMP type I receptor complexes [22–24], BMPR-IA is bound to the so-called wrist epitope of GDF-5 formed by the finger region of one GDF-5 monomer and the helix and pre-helix loop of the second GDF-5 monomer subunit [PDB:3QB4] [24]. Although a full complex—one GDF-5 R57A dimer and two BMPR-IA_EC_ molecules (Fig. 1a, b)—was observed in the asymmetric unit of the crystal, the backbone of the ligand–receptor complex was highly symmetrical, as shown from the superposition of the two GDF-5 monomers as well as the BMPR-IA ectodomains (Additional file 1: Figure S1). Analysis of the two binding interfaces formed by the homodimeric GDF-5 and the two BMPR-IA molecules yielded a total of 18 intermolecular hydrogen bonds (H-bonds), suggesting that polar bonds play a role in GDF-5 type I receptor recognition and binding (Table 1). However, because the 18 H-bonds were formed from 12 pairs of polar groups, the H-bond network was not identical in the two otherwise identical interfaces (Table 1). In interface 1 of the GDF-5 R57A:BMPR-IA_EC_ complex, 11 H-bonds were found between the ligand and the receptor, whereas in interface 2, only seven H-bonds were observed (Table 1). A comparison showed that six H-bonds were identically formed in both interfaces with highly similar length and angles, therefore potentially representing the physiologically important core of the polar interactions.

**Table 1:** Geometry of H-bonds in the GDF-5 R57A:BMPR-IA_EC_ interface

GDF-5 R57A	BMPR-IA_EC_	Distance Å	Angle N–O–C^a^	H-bond^b^
R18 (NE)	G42 (O)	3.19 (−)	162 (−)	SC-MC
W33 (NE1)	D89 (O)	2.94 (−)	133 (−)	SC-MC
L56 (N)	Q86 (OE1)	2.88 (2.81)	140 (143)	MC-SC
L56 (O)	Q86 (NE2)	3.12 (3.19)	137 (140)	MC-SC
S58 (N)	C77 (O)	2.74 (2.86)	129 (130)	MC-MC
S58 (OG)	T55 (OG1)	2.93 (2.54)	99 (121)	SC-SC
E61 (OE2)	K79 (NZ)	3.26 (−)	125 (−)	SC-SC
M31 (O)	S90 (OG)	2.55 (−)	136 (−)	MC-SC
S74 (O)	Q94 (N)	2.83 (3.03)	149 (142)	MC-MC
S74 (OG)	R97 (NH2)	- (2.87)	- (132)	SC-SC
K107 (NZ)	D84 (OD2)	3.01 (−)	117 (−)	SC-SC
Y109 (OH)	D84 (OD2)	2.61 (2.62)	132 (139)	SC-SC
Mean value		2.94 (2.84)	131/139 (135/136)	
S.D.		0.21 (0.22)	10 (13)	

A total of 18 intermolecular H-bonds were found in the two interfaces between the GDF-5 dimer and the two BMPR-IA receptors. These non-covalent interactions were formed between 12 atom pairs resulting in an uneven distribution of the 18 H-bonds. The numbers in parentheses are the distances between donor and acceptor atoms and N–O–C angles in the second interface, related by non-crystallographic symmetry

^a^N, O, C are the donor-acceptor atoms; from general statistics^20^ this angle is 149 ° ±15 ° for MC–MC hydrogen bonds and 129 ° ±18 ° for SC–MC and SC–SC H-bonds

^b^MC (main chain) and SC (side chain) donor/acceptor atoms

### GDF-5 undergoes an induced fit mechanism upon binding to type I receptors

**Fig. 2:**
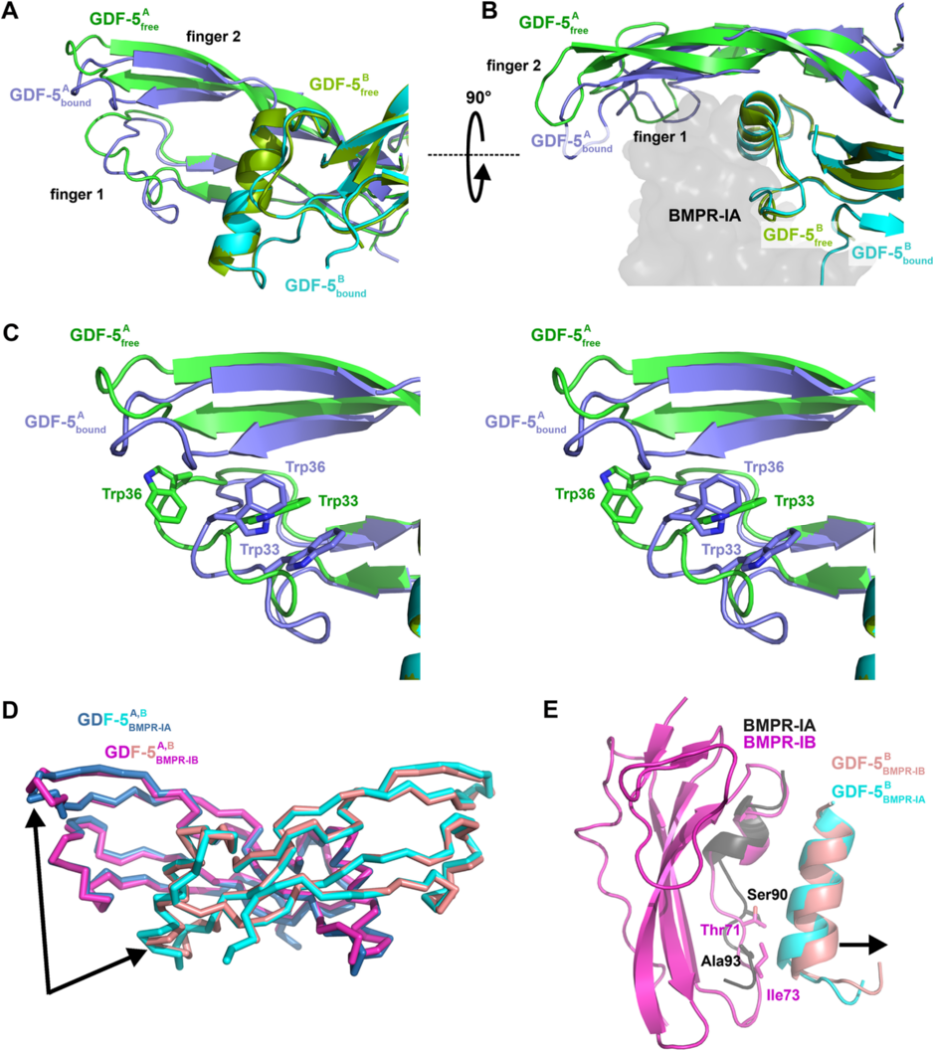
Conformational rearrangement of GDF-5 upon complex formation. **a** Structural alignment of unbound (*green*) and GDF-5 R57A (*blue/cyan*) bound to BMPR-IA showing an induced fit rearrangement of the tips of finger 1 and 2 upon type I receptor binding. **b** As in (**a**) but viewed from the top. Upon binding to BMPR-IA, finger 1 and 2 of GDF-5 move towards the BMP type I receptor surface (shown as transparent van der Waals surface representation). **c** Stereoview of the conformational change of GDF-5 upon type I receptor binding. The side chain of Trp33 and Trp36 show the largest rearrangement, moving up to 10 Å. **d** Structural alignment of GDF-5 (wild-type) bound to BMPR-IB (*magenta*/*rose*) and GDF-5 R57A (*blue*/*cyan*) bound to BMPR-IA showing that the bound conformation of GDF-5 is almost identical irrespective of the type I receptor bound. **e** Different spatial requirements due to amino acid differences between BMPR-IA (*black*, only residues Glu81 to Gln94 are shown) and BMPR-IB (*magenta* ribbon plot) result in a small but significant bending of the α-helix 1 of GDF-5 (GDF-5 bound to BMPR-IA shown in *cyan*, GDF-5 bound to BMPR-IB colored in *rose*)

For GDF-5, superposition of the free [PDB:1WAQ] [19] and bound structures yielded an root mean square (r.m.s.) deviation of 1.7 Å for all Cα-atom positions, but significant differences were mainly observed for the tips of fingers 1 and 2 (Fig. 2a, b). Upon binding of GDF-5 R57A to BMPR-IA_EC_, the backbone atoms of residues Phe97 to Val104 (finger 2) and Lys29 to Ile38 (ω-loop in finger 1) of GDF-5 R57A moved up by 5.5 Å towards BMPR-IA, although residues in the very tips of finger 2 shared no contact with BMPR-IA. With regards side chain rearrangements, the largest changes were observed for the two tryptophans in the wrist epitope of GDF-5 R57A (Fig. 2c); Trp33 moved downwards by about 4 Å and Trp36 rotated from a highly solvent-exposed conformation into a position located above Trp33 thereby moving by almost 10 Å. Residues in the α-helix showed no conformational changes upon complex formation and residues in the pre-helix loop of GDF-5 R57A, the latter of which is considered to be rather flexible in BMP-2 [22], shifted by 2.2 Å (residues Phe54 to Pro55) at the N-terminal start of the pre-helix loop. A comparison of the complexes of GDF-5 R57A bound to BMPR-IA_EC_ and wild-type GDF-5 bound to BMPR-IB_EC_ [PDB:3EVS] [24] revealed that the structures of “bound” GDF-5 were highly similar irrespective of what type I receptor was bound (Fig. 2d). The observation indicates that the induced fit of GDF-5 upon complex formation, e.g., the “closing” of the two finger tips, possibly follows a route independent of the exact nature of the type I receptor. Thus GDF-5 seems to “snap” into an imprinted bound conformation upon binding to a type I receptor. The only small differences in the backbone conformations of GDF-5 and GDF-5 R57A bound to either BMPR-IA_EC_ or BMPR-IB_EC_ were observed for residues Ser74 to Thr80, covering the last turn of the α-helix and the loop in front of β-strand 5 of finger 2. Owing to differences in the structures of the contacting loops of BMPR-IA and BMPR-IB, these residues shifted by 1.3–2.6 Å; in GDF-5 in complex with BMPR-IB_EC_, this region was pushed away from the GDF-5 surface to provide more space for several bulkier amino acid residues in BMPR-IB (e.g., Thr71 BMPR-IB versus Ser90 BMPR-IA; Ile73 BMPR-IB versus Ala93 BMPR-IA) (Fig. 2e).

### The receptor BMPR-IA adopts a similar conformation when bound to different BMP ligands

**Fig. 3:**
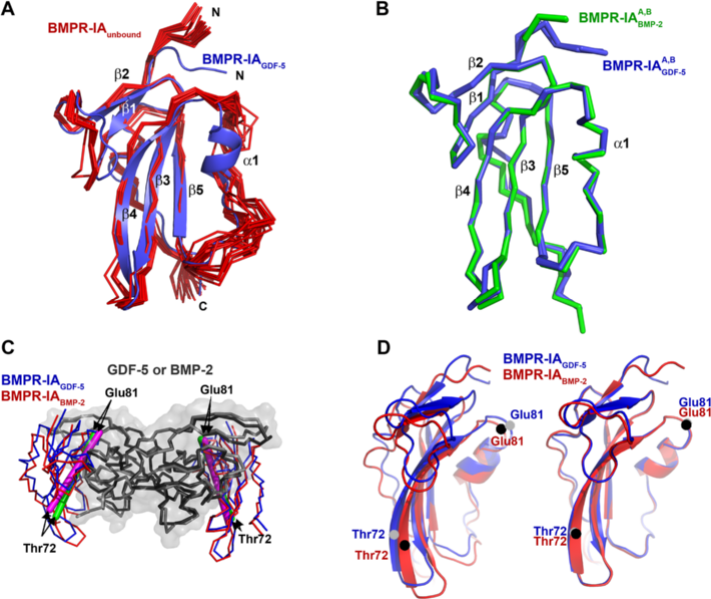
Ligand-dependent reorientation of the receptor ectodomain. **a** Comparison of the structure of unbound BMPR-IA, the structure ensemble comprising 21 structures [PDB:2K3G] is shown as a *red* Cα-trace, and BMPR-IA bound to GDF-5 R57A (*blue*). **b** Structure alignment of BMPR-IA bound to GDF-5 R57A (*blue*) and bound to BMP-2 (*green*). The Cα-traces of both BMPR-IA structures overlap almost perfectly; minor conformational differences of the backbone are only observed for the β3β4-loop and the C-terminus. **c** Superposition of the complexes GDF-5 R57A:BMPR-IA and BMP-2:BMPR-IA reveals that the BMPR-IA ectodomains adopt slightly different orientations as illustrated by lines running through the Cα-atoms of Glu81 and Thr72 (*magenta line* BMPR-IA of the complex GDF-5 R57A:BMPR-IA; *green line* BMPR-IA of the BMP-2:BMPR-IA complex, the Cα position of either Thr72 or Glu81 is indicated by *arrows*). **d** Magnification of the two BMPR-IA molecules bound to GDF-5 R57A or bound to BMP-2 showing the different tilt angle of the BMPR-IA moieties in both ligand–receptor complexes. The Cα-atom positions of residues Thr72 and Glu81 in the BMPR-IA molecules are indicated by *black circles* (BMPR-IA from the complex when bound to BMP-2) and *gray circles* (BMPR-IA from the complex when bound to GDF-5 R57A) showing the differing orientation of both BMPR-IA molecules in the complex. Superpositioning of both BMPR-IA moieties shows that the orientational difference is not due to ligand-induced different conformations within the BMPR-IA molecules

For BMPR-IA, structure determination by nuclear magnetic resonance and comparison with its structure bound to BMP-2 revealed a large induced fit upon complex formation with BMP-2 [25]. The β4β5-loop of BMPR-IA, which is the central element of the BMP receptor–ligand interaction, was disordered in the “free” BMPR-IA, whereas the conformation of this loop was constrained when bound to BMP-2 and contained a short 1.5-turn helix (Gly82–Asp89). In complex with GDF-5 R57A, the α-helix of BMPR-IA formed similarly as seen in the complex with BMP-2, consistent with the observation in the study of Klages et al. [25] that small changes in the environment result in spontaneous α-helix formation (Fig. 3a). Also, a similar intermolecular H-bond network emanated from residues located within the α-helix of BMPR-IA_EC_ involving the backbone of Leu56 of GDF-5 R57A to the carboxamide group of BMPR-IA Gln86 (Table 1). The aromatic side chain of Phe85 of BMPR-IA made similar hydrophobic “knob into hole” contacts with GDF-5 R57A as seen in BMP-2:BMPR-IA_EC_. Importantly, because the complex of BMPR-IA_EC_ bound to GDF-5 R57A represented the first structure of BMPR-IA_EC_ bound to a BMP ligand other than BMP-2, we were able to compare the influence of different ligands on the structure of BMPR-IA and analyze whether the promiscuous binding of BMP receptors to various ligands is due to structural plasticity in their binding site.

Superimposing BMPR-IA bound to GDF-5 R57A or BMP-2 showed an almost identical conformation for BMPR-IA_EC_ as evident from an r.m.s. deviation of only 0.7 Å for the Cα atoms of residues Pro34 to Pro117 (Fig. 3b). None of the Cα-atom positions in the BMPR-IA molecules bound to either GDF-5 or BMP-2 differed by more than 1 Å. Even when all heavy atoms were considered, significant differences were only observed for side chains located at the solvent accessible surface of BMPR-IA outside the binding site (r.m.s. deviation 1.5 Å for Pro34 to Pro117). Because BMPR-IA_EC_ seemed to adopt the same structure irrespective of whether it was bound to GDF-5 R57A or BMP-2, alterations in the receptor structure required for the adaptation to different ligand surfaces might be minimized by small concomitant adaptations in the ligand and the receptor.

### Orientation of the BMPR-IA ectodomain differs among BMP ligand-receptor complexes

Although the structures of the ligands as well as of BMPR-IA were almost identical in the different complexes with varying binding partners, superposition of the complexes of GDF-5 R57A:BMPR-IA and BMP-2:BMPR-IA revealed that the orientation/position of the receptor ectodomain in its binding site differed between these complexes (Fig. 3c). This became evident when the Cα-atoms of both ligand molecules were superimposed, showing that the butterfly-like architecture of the BMP-2 and GDF-5 backbone overlapped almost perfectly. Distances between the Cα-atoms of Glu81 of both BMPR-IA molecules in either GDF-5 R57A:BMPR-IA or BMPR-2:BMPR-IA, which was located at the membrane-distal part of the receptor epitope, then showed differences of 2.3 Å with the shorter distance present in GDF-5 R57A:BMPR-IA. Similarly, measuring the distance between the Cα-atoms of Thr72, which was located at the membrane-proximal part of BMPR-IA, revealed a difference of 2.6 Å when both complexes were compared. The Cα-atom of Gln86 could be considered as a center of rotation for the BMPR-IA molecule. Because the above-mentioned distances reflect distances above and below the center of rotation, the differences observed between both complexes indicated that the BMPR-IA moieties were exhibiting a different tilt angle with respect to each other as well as with respect to the ligand (Fig. 3c, d). Indeed, when defining a line through the Cα-atoms of Thr72 (β-strand 4) and Glu81 (C-terminal end of β-strand 4) at the edges (or a plane by additionally using Glu64 in β-strand 3) and calculating the angles between the lines/planes of the two BMPR-IA molecules in both BMP receptor complexes, the tilting of the two receptor moieties differed by about 10 °. From this comparison we determined that the membrane-distal part of the BMPR-IA molecules swiveled towards the N-terminal end of the GDF-5 R57A α-helix and further into the concave wrist epitope, whereas the membrane-proximal half of the receptor swung out of the binding site compared to the receptor orientation observed for the BMP-2:BMPR-IA complex. It seems that this different receptor orientation/tilting depends on the nature of the ligand, because redoing the analysis with GDF-5 R57A:BMPR-IA and GDF-5:BMPR-IB yielded a similar receptor tilting with less than 1 ° difference. This observation might explain the lack of structural plasticity seen in the structures of BMPR-IA_EC_ bound to GDF-5 or BMP-2. Instead of the BMPR-IA receptor ectodomain adopting different conformations to adapt to variations in the surface geometries of GDF-5 and BMP-2, the altered tilting of the receptor accommodates differences in the partner’s binding site geometry.

### Specific recognition of the type I receptors BMPR-IA and BMPR-IB due to a spring-loaded latch in GDF-5

**Fig. 4:**
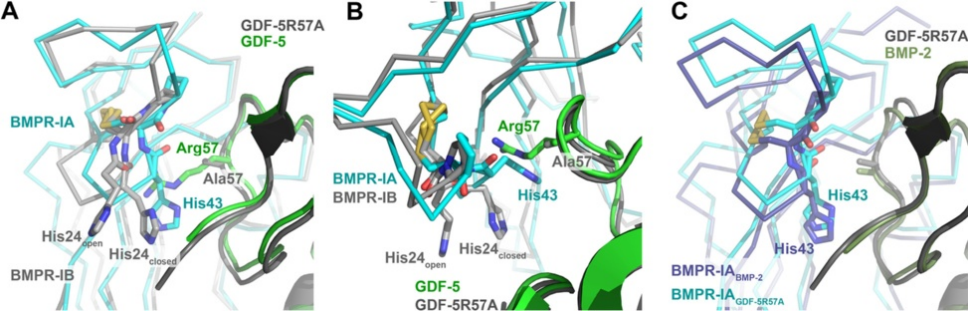
BMP type I receptor discrimination by GDF-5. **a** The β1β2-loop of the BMP type I receptor BMPR-IA (Cα-trace in *cyan*) and BMPR-IB (*gray*) adopt different conformations. Two alternative conformations are observed for this loop in the complex GDF-5:BMPR-IB; in contrast, the β1β2-loop of BMPR-IA bound to GDF-5 R57A adopts a single locked conformation that is impossible if a large arginine side chain is at position 57 (as for example in GDF-5WT). **b** As in (**a**) but rotated around the y-axis by 90 ° showing the putative steric clash between the side chains of His43 (*cyan*) in the β1β2-loop of BMPR-IA and Arg57 of wild-type GDF-5 (*green*). **c** The β1β2-loop of BMPR-IA (*cyan*) bound to GDF-5 R57A and BMPR-IA (*blue*) bound to BMP-2 adopt identical backbone and side-chain conformations

On a molecular level, discrimination of GDF-5 between the two type I receptors is likely due to different conformations of the β1β2-loop in the receptors, thereby generating or releasing steric hindrance. In the crystal structure of GDF-5 bound to BMPR-IB_EC_, the β1β2-loop is in an open conformation, providing enough space for the bulky side chain of Arg57 of GDF-5 [24] (Fig. 4a, b; Additional file 2: Figure S2). Modeling of the complex of GDF-5 bound to BMPR-IA_EC_ using the structure of BMP-2:BMPR-IA_EC_ [PDB:1REW] [22] suggested that the loop of BMPR-IA might adopt a “closed” conformation, but the differences observed in the receptor tilting between the complexes GDF-5:BMPR-IB_EC_ and BMP-2:BMPR-IA_EC_ did not allow for a definite conclusion. The structure of the GDF-5 variant R57A bound to BMPR-IA_EC_, however, proved this theory to be correct. The β1β2-loop conformation of the receptor BMPR-IA_EC_ was identical in both complexes, with BMPR-IA bound to either GDF-5 R57A or BMP-2 (Fig. 4c). Thus with the β1β2-loop of BMPR-IA_EC_ adopting the same closed conformation as observed in the complex BMP-2:BMPR-IA_EC_, residue His43 in the β1β2-loop of BMPR-IA would clash with Arg57 of GDF-5 (Fig. 4a, b). To release this steric hindrance either the β1β2-loop has to change its backbone conformation or the side chain of Arg57 of GDF-5 has to reorient. Because Arg57 is tightly packed by surrounding residues from GDF-5 as well as residues from the receptor BMPR-IA, it is more likely that the β1β2-loop of BMPR-IA reorients to create space for Arg57.

### GDF-5 inhibits BMP-2 induced ALP expression in C2C12 cells

**Fig. 5:**
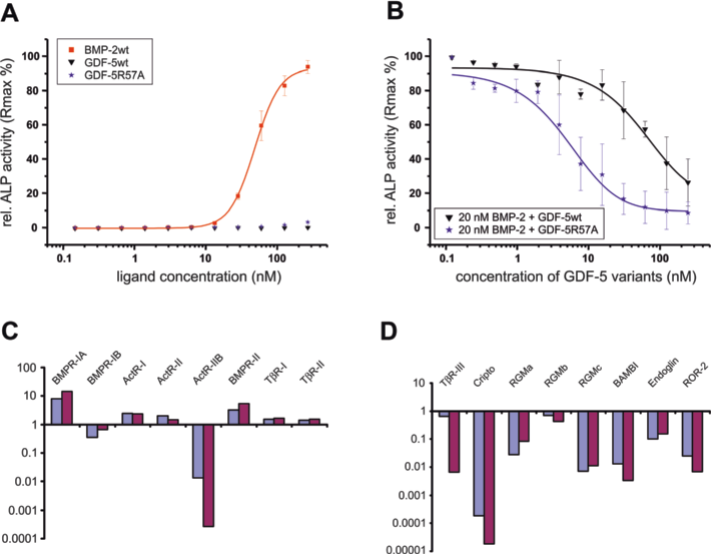
Ligand-mediated induction of alkaline phosphatase (*ALP*) expression. **a** Dose-dependent induction of ALP gene expression in C2C12 cells. The background absorption signal of 0.08 ± 0.004 at 405 nm was subtracted from all data points. The ALP activity induced by 250 nM of BMP-2 was normalized to 100 %. **b** Inhibition of BMP-2-induced ALP expression by increasing concentrations of GDF-5 proteins. Half-maximal inhibitory concentrations (IC_50_) of 70 nM and 5 nM were observed for wild-type GDF-5 (*wt*) and GDF-5 R57A, respectively. Expression levels (mRNA) of receptors (**c**) and co-receptors (**d**) in C2C12 (*blue bars*) and ATDC-5 (*purple bars*) cells were determined by real-time reverse transcription polymerase chain reaction. Bars represent mean values of two individual experiments. All values were normalized to *HPRT* gene expression. (For primer sequences and statistics see Additional file 4: Table S1)

BMP-2 can induce ALP expression in ATDC-5 and in C2C12 cells with similar efficacy [18, 19, 26]. In contrast, wild-type GDF-5 as well as the variants R57L [18] and R57A (Fig. 5a; Additional file 3: Figure S3) exhibited significant induction of ALP only in ATDC5 cells. For the two GDF-5 variants this result was rather unexpected. Their binding affinities to all type I and type II receptors are highly comparable if not identical to BMP-2, and it was thus expected that, owing to their BMP-2-mimicking receptor binding properties, BMP-2-like activities would be observed [17]. To directly compare the biological activities of the ligands, ALP assays using C2C12 cells were performed (Fig. 5a). To test whether GDF-5 or GDF-5 R57A can compete for BMP-2-mediated ALP induction, and thus indeed utilize the same BMP receptors as BMP-2, the cells were treated simultaneously with 20 nM of BMP-2 and increasing concentrations of GDF-5 or the variant R57A (Fig. 5b).

The direct comparison of the three ligands revealed clear differences in their ALP-inducing potential. Addition of up to 250 nM of GDF-5 did not increase ALP levels in these cells, and GDF-5 R57A showed only slightly increased ALP activities even at high concentrations despite being considered a BMP-2 mimic (Fig. 5a). Instead, a competition assay revealed a dose-dependent reduction of BMP-2-induced ALP activity upon simultaneous addition of either one of the respective GDF-5 ligands (Fig. 5b). The inhibitory effect of the R57A variant (IC_50_ 5 nM) was ~14-fold stronger compared to that of wild-type GDF-5 (IC_50_ 70 nM), which directly correlates with the increased affinity of GDF-5 R57A for BMPR-IA. It is important to note that the IC_50_ values determined here were basically identical to the EC_50_ values observed for the GDF-5 ligands in ALP assays using ATDC-5 cells (Additional file 3: Figure S3). Accordingly, the inhibitory function seems most likely to result from competition for a common receptor-binding site present in both ATDC-5 and C2C12 cells. But, based on the cellular context, both GDF-5 variants act either as an agonist or an antagonist.

### Expression profiles of BMP receptors and co-receptors are highly similar in ATDC-5 and C2C12 cells

To investigate whether these opposing GDF-5 activities result from differences in the expression profile of BMP receptors or known co-receptors, quantitative reverse transcription polymerase chain reaction (qRT-PCR) experiments were performed using mRNA libraries derived from non-stimulated ATDC-5 and C2C12 cells (Fig. 5c, d; for primer sequences and statistics see Additional file 4: Table S1). The expression levels for each BMP receptor type were highly similar in ATDC-5 and C2C12 cells (difference below 2-fold), except for ActR-IIB. In the case of known co-receptors, only the TβR-III receptor (betaglycan) was differentially expressed, with levels 100 times higher in C2C12 cells. This co-receptor was recently shown to act as a BMP cell surface receptor, and direct and specific binding of this receptor to BMP-2, BMP-4, and BMP-7 (and to a lesser amount to GDF-5) could be demonstrated in cross-linking experiments [27]. To test a possible modulation of GDF-5 signaling by this receptor in ATDC-5 cells, we transfected these cells with a TβR-III expression plasmid (kindly provided by P. ten Dijke, Leiden) and analyzed GDF-5-mediated ALP induction. However, a comparison of transfected and non-transfected cells revealed no difference upon GDF-5 stimulation (Additional file 5: Figure S4A). Similarly, transfection of ActR-IIB into ATDC-5 cells did not alter GDF-5 responsiveness (Additional file 5: Figure S4B).

### Ligand-induced ALP expression is mediated via the type I receptor BMPR-IA

**Fig. 6:**
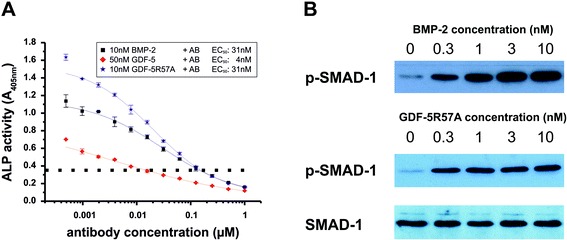
Inhibition of ligand-induced signal transduction. **a** Dose-dependent inhibition of ALP gene expression induced by BMP-2 (*black squares*), GDF-5 (*red diamonds*), or GDF-5 R57A (*blue asterisks*) by increasing concentrations of the BMPR-IA-neutralizing Fab antibody AbD1564. The background absorption signal (without ligand, *dotted line*) of 0.36 ± 0.04 at 405 nm was not subtracted to highlight ALP induction mediated by endogenously expressed BMP ligands. Half-maximal inhibitory antibody concentrations (IC_50_) for ALP activities induced by the different ligand proteins are indicated. A biological duplicate of this experiment is shown in Additional file 6: Figure S5. **b** Ligand-induced SMAD-1 phosphorylation in C2C12 cells. The cells were serum-starved and exposed to the indicated ligands for 1 h. Equal amounts of whole cell lysates were separated by SDS-PAGE and western blots were performed using an anti-SMAD-1 or anti-pSMAD-1 antibody

In our qPCR experiments, *bmpr-1a* transcripts showed the highest abundance, whereas those of *bmpr-1b* were found to have at least 10-fold lower expression in both cell lines. However, because the number of receptors present at the cell surface does not necessarily correlate with the mRNA levels measured, we performed ALP assays with ATDC-5 cells in which signaling through BMPR-IA was blocked by a highly specific BMPR-IA-neutralizing Fab antibody (AbD1564, AbD Serotec, Puchheim, Germany). When applied with both factors used here, the neutralizing antibody caused a dose-dependent reduction of ALP activity (Fig. 6a; Additional file 6: Figure S5). Most importantly, the signaling derived from application of exogenous BMP-2 and GDF-5 was quantitatively abolished upon application of high antibody concentrations because ALP activity was attenuated even below background levels (i.e., levels without ligand addition pointing to the presence of endogenous BMPR-IA-binding TGF-β family ligands). The differences in the antibody concentrations required for half-maximal inhibition (IC_50_) of either BMP-2, GDF-5, or its variant R57A directly correlate with their binding affinities for the receptor BMPR-IA [17]. The affinity of the neutralizing antibody for BMPR-IA (*K*_D_ = 2.2 nM) is higher than that of GDF-5 for BMPR-IA (*K*_D_ = 20 nM) but slightly lower than the receptor affinities of BMP-2 (*K*_D_ = 0.8 nM) or GDF-5 R57A (*K*_D_ = 1.6 nM). In summary, our data clearly identified BMPR-IA as the sole type I receptor mediating BMP-2-induced as well as GDF-5-induced ALP activity in ATDC-5 cells.

### Antagonistic activities of GDF-5 are not related to signal transduction via the SMAD pathway

Owing to the similar receptor and co-receptor expression pattern in ATDC-5 and C2C12 cells, we tried to unravel why C2C12 cells do not express ALP in response to GDF-5 and, furthermore, how GDF-5 can act as a BMP-2 antagonist in this cell line. One possible mechanism for a GDF-5-mediated antagonism might be related to a sequential order of the receptor recruitment and activation [13]. Because GDF-5, similar to BMP-2, binds first to the type I receptor BMPR-IA, recruitment of the type II to this intermediate GDF-5:BMPR-IA complex and/or the subsequent activation of BMPR-IA might then be impeded in C2C12 cells, explaining the difference in signaling. We therefore tested whether addition of wild-type GDF-5 or the BMP-2 mimic GDF-5 R57A leads to the formation of signaling-competent receptor complexes by analyzing the phosphorylation status of SMAD molecules, given that this signaling cascade directly depends on ternary ligand–receptor complex formation (Fig. 6b).

In contrast to ALP expression, which in C2C12 cells was only induced by BMP-2, SMAD-1 phosphorylation was observed after exposure to BMP-2, GDF-5, and GDF-5 R57A at similar levels in both ATDC5 and C2C12 cell lines. The signals were stable over a period of 72 h (Additional file 7: Figure S6), as published for BMP-2 previously [17]. For wild-type GDF-5, about 10-fold higher concentrations were required to achieve phospho-SMAD levels comparable to those resulting from BMP-2 stimulation, which again directly correlates with the affinity differences for BMPR-IA binding of GDF-5 and BMP-2. Because ternary ligand–receptor complex formation is an essential prerequisite for SMAD activation, but is observed here in ALP-positive ATDC-5 as well as in ALP-negative C2C12 cells, cell-specific differences in the recruitment of the type I and type II receptors by GDF-5 as compared to BMP-2 seem highly unlikely. Because the p38 MAP kinase pathway has been previously described in the context of GDF-5 as well as in BMP-2 signaling [28–30], we then investigated p38 MAP kinase levels and activation upon GDF-5, GDF-5 R57A, and BMP-2 stimulation. ATDC-5 cells exhibited lower p38 MAP kinase expression compared to C2C12 cells, and only the latter showed p38 MAP kinase activation upon treatment with the different BMP proteins. However, no significant differences could be observed between the individual factors (Additional file 7: Figure S6). Thus, the data presented here strongly point to additional component(s) in either ATDC-5 or C2C12 cells, which either facilitate ALP induction by GDF-5 in the former or suppress GDF-5-mediated ALP induction in the latter cell line.

### Agonist and antagonist activities of GDF-5 are also observed in vivo

**Fig. 7:**
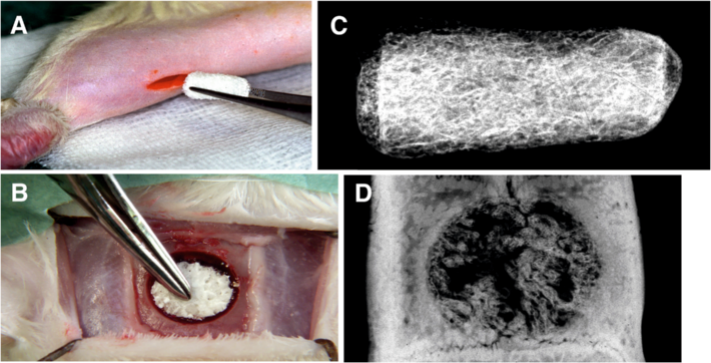
Ligand implantation assay in rat. Insoluble collagenous bone matrix cylinders doped with BMP-2, GDF-5, or GDF-5 R57A were implanted into a hind-limb muscle (**a**) or the calvaria (**b**) of male adult Sprague Dawley rats with a body weight of 400 ± 50 g. The cylinders were explanted 28 days post implantation and radiographs of the specimen were taken. Bone regeneration was assessed by quantifying the area of newly formed bone of the total explant (**c**) or in the defect site (**d**)

The C2C12 cell line was originally obtained from muscular tissue from mice [31] and its specific pre-myoblastic properties, e.g., their potency to differentiate into multinuclear myotubes upon serum starvation, are maintained. Addition of certain subset of BMPs leads to differentiation towards the osteoblastic lineage. This cell line therefore resembles somewhat the situation in muscular tissues of animals in which ossicles form ectopically upon BMP-2 implantation. To compare the biological activities of the ligands observed in our cell-based assays with data from an in vivo model, carriers were doped with ligands and implanted into the hind limbs of rats. Additionally, the ligand proteins were also implanted at an orthotopic site in order to get information on their osteoconductive properties (Fig. 7).

**Fig. 8:**
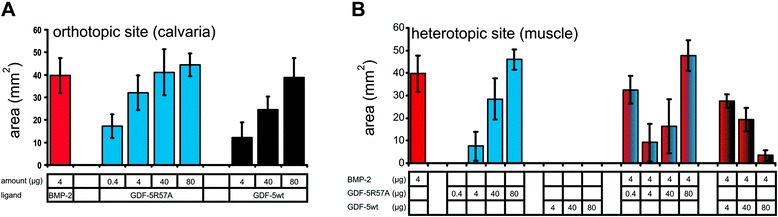
Ligand-induced bone formation in vivo. Quantification of the ossified surface areas induced by the indicated amounts of ligands doped on the implanted insoluble collagenous bone matrix cylinders. The data represent mean values of explants from at least five individual animals. **a** Orthotopic model (calvaria); **b** heterotopic model (hind-limb muscle). For statistics, see Additional file 8: Table S2 and Additional file 9: Table S3

Analyses of these experiments provided quantitative data on the osteoconductive properties of all ligand proteins tested in the orthotopic model (Fig. 8a, for statistics see Additional file 8: Table S2). Using 4 μg of BMP-2 resulted in bone formation corresponding to about 40 mm^2^ surface area and served as a reference for the other ligands. Implanting 4 μg of GDF-5 R57A resulted in a slightly smaller amount of bone formation. In contrast, wild-type GDF-5 required about 20-fold more protein than BMP-2 or GDF-5 R57A to obtain ossification of the same surface area. These results fully correspond with the ALP-inducing properties of these ligand proteins in ATDC-5 cells [18, 19] and also correlate with the in vitro affinities of these ligands for BMPR-IA. Based on the attenuated osteoconductive potential of wild-type GDF-5 in the calvaria, we assume that this process—as for the induction of ALP expression in ATDC-5 cells—involves the type I receptor BMPR-IA.

Upon implantation of the ligands at the heterotopic site (here: muscle) we observed a very different picture (Fig. 8b, for statistics see Additional file 9: Table S3). The ability of BMP-2 to induce the formation of ectopic bone at heterotopic sites has long been known [32], whereas these properties are controversial in GDF-5. Recently, Kakudo and colleagues reported that GDF-5 induced the formation of ectopic bone in rat leg muscles, but they used an excessive dose of more than 300 μg of wild-type GDF-5 [33]. In comparison, different reports have shown that 2 μg or less of recombinant BMP-2 can induce ectopic bone growth [34–36]. In our experiments, a direct comparison of the ligand proteins after implantation at heterotopic sites showed that ectopic bone formation was indeed induced by BMP-2 as well as by GDF-5 R57A. However, 10-times more of the BMP-2 mimic GDF-5 R57A protein was required to induce bone formation of a similar degree, despite its receptor binding properties being identical to those of BMP-2. Wild-type GDF-5 did not induce any bone formation even at very high doses (80 μg). Even more surprising, application of a combination of BMP-2 with increasing amounts of GDF-5 R57A first led to a clear reduction in bone formation compared to application of BMP-2 alone (see Fig. 8b columns 13 and 14, 4 μg BMP-2 plus 4 μg GDF-5 R57A, and 4 μg BMP-2 plus 40 μg GDF-5 R57A). This observation strongly indicates that BMP-2 and GDF-5 R57A utilize the same BMP receptors in this in vivo environment because the presence of GDF-5 R57A led to direct attenuation of BMP-2 activity. Consistently, wild-type GDF-5—showing no bone formation up to amounts of 80 μg—could also efficiently antagonize BMP-2 activity in a clear dose-dependent manner. Because GDF-5 R57A binds BMPR-IA with about 10–20-fold higher affinity than wild-type GDF-5, the variant could also block BMP-2-induced bone formation more efficiently (see Fig. 8b columns 13 and 19, 4 μg BMP-2 plus 4 μg GDF-5 R57A, and 4 μg BMP-2 plus 80 μg GDF-5). These observations in vivo somewhat resemble the BMP-2 antagonizing properties of GDF-5 and its variant found in the in vitro ALP-induction experiments in C2C12 cells (see Fig. 5b).

However, when larger amounts of GDF-5 R57A were applied together with BMP-2, the intrinsic activity of the GDF-5 variant (Fig. 8b, column 6, 80 μg GDF-5 R57A) then resulted in bone formation similar to that seen for GDF-5 R57A alone, but no synergistic or additive effects were observed when both osteoconductive factors were simultaneously applied. This suggests that GDF-5 R57A, although recruiting the same BMP type I and type II receptors as BMP-2 and doing so with the same affinities, exhibits a dose-dependent activity distinct from BMP-2 in this cellular environment. Combining the observations from the in vivo and cell-based in vitro studies indicates that, dependent on the cellular context and with respect to certain cellular signals, GDF-5 can act either as an agonist or antagonist of BMP-2, which might have important implications in processes where both factors act simultaneously on a heterogeneous population of cells, as would be the case, for example, in the developing joint.

## Discussion

An intriguing feature of the TGF-β superfamily is the limited specificity of its ligand–receptor interactions arising from the numeral discrepancy of sharing only seven type I and five type II receptors among the more than 30 ligands identified in mammals to date. A further reduction in the signaling variety—disregarding cross talks with other pathways—is due to the fact that this large number of ligands principally activates only two different SMAD signaling pathways. This strong signal convergence raises important questions on how these proteins can then encode so many different cellular (and ligand-specific) functions.

Structural analyses of different ligands in complex with the type I receptors BMPR-IA and BMPR-IB have revealed differences in the orientation of the type I receptors in these complexes. A comparison of the complexes BMP-2:BMPR-IA_EC_ and GDF-5:BMPR-IB_EC_ showed an altered tilting between the two receptor moieties in the complex by 20 ° (10 ° for each receptor ectodomain), with the membrane-distal part of BMPR-IB_EC_ moving into the wrist epitope and the membrane-proximal part moving out of the wrist epitope of GDF-5 [24]. However, because complexes of two different ligands bound to different type I receptors were compared here, the nature and significance of this change in ligand–receptor architecture remained unclear. With the structure of GDF-5 R57A bound to BMPR-IA_EC,_ we have here been able to compare these differences, and found that the change in tilting of the two type I receptor moieties seems to be ligand-specific. The type I receptors BMPR-IB and BMPR-IA share almost identical (within 1 °) tilt angles in the complexes GDF-5:BMPR-IB_EC_ and GDF-5 R57A:BMPR-IA_EC_, whereas both complexes show a similar difference compared to the orientation of BMPR-IA_EC_ in the complex BMP-2:BMPR-IA_EC_. Although this change in type I receptor orientation may seem small, it might nevertheless contribute to a different signaling output for both factors. Furthermore, the structure of another BMP ligand–receptor complex, BMP-9 bound to the type I receptor Alk1 and the type II receptor ActR-IIB, has been determined [37], and shows an even stronger tilting of the type I receptor extracellular domain Alk1 in the complex, suggesting that the ligand–receptor architecture differs for different BMP ligands. This architectural variance might provide a possible mechanism for ligand-specific signals even when using overlapping sets of receptors if the orientation difference can be transmitted across the membrane.

For receptors with a single transmembrane helix, receptor activation was originally assumed to occur via ligand-induced receptor dimerization or oligomerization as determined for the human growth hormone (hGH) [38]. This simplistic activation mechanism, relying purely on dimerization/oligomerization of two receptor subunits, thereby bringing intracellular domains into close proximity, has been challenged in the past 10 years by several studies on typical receptor family members such as the erythropoietin (EPO) and hGH receptors. The first surprising data, from Livnah et al., showed that EPO mimetics (EMP) forming symmetrical EMP:(EPOR)_2_ ligand–receptor complexes can activate the receptor, but that mimetics forming an asymmetric EPO receptor complex, deviating from a 180 ° symmetrical dimer by 15 °, remained inactive [39]. Although this orientation-dependent activation hypothesis for single transmembrane receptors was initially debated because of the suggestion that other properties of this EPO mimetic could result in loss of activity [40], subsequent studies supported that activation of single transmembrane receptors might be beyond a ligand-induced encounter of the cytoplasmic domains. That (proper) receptor–receptor orientation plays an important role in downstream signaling transduction was confirmed by three elegant experiments. One used a set of monoclonal antibodies that could arrange two receptors in different dimer assemblies [41]; another used coiled-coil domains as ligand replacements and altered their dimer architecture [42]; and a third introduced or deleted residues within the transmembrane helix to alter the helical pitch and relative orientation of the two receptor subunits in the dimer arrangement [43].

The above-depicted mechanism provides a potential scenario for how two different ligands such as GDF-5 and BMP-2 might generally encode different signals via an identical receptor assembly, and is helpful in explaining the functional diversity of BMP ligands on the whole. However, it cannot explain the difference in the signaling characteristics of GDF-5 (or its variant R57A) observed here between the two cell lines C2C12 and ATDC5 or between the heterotopic and orthotopic ossification model, because we have to assume that the ligand-specific differences in the receptor architecture are identical in both cell lines and in both tissue environments.

Another explanation of why the ossification potency of GDF-5 and BMP-2 differed in the in vivo experiments might be the different release of both growth factors from the carrier (resulting from different diffusion/solubility properties). However, one would then expect similar results in both ossification models, because the carriers used in both models were identical. Furthermore, diffusive properties of the mature form of BMPs seem to be regulated by ECM binding sites usually formed by a stretch of positively charged residues present in the N-terminus of these factors [44]. The N-terminal regions of GDF-5 and BMP-2 are, however, similar in length and contain a comparable number of basic residues, likely indicating similar ECM binding properties.

What potential mechanisms could then lead to cell type-specific and ligand-specific signaling differences for GDF-5 and enable GDF-5 to act as a context-dependent antagonist against BMP-2? Possibly the simplest explanation without considering thus far unknown components would be the formation of heteromeric receptor assemblies in a cell type-dependent manner. But GDF-5 and BMP-2 exhibit no ligand-specific affinity differences for their three type II receptors [17]: a heteromeric receptor assembly with a ligand-specific type II receptor stoichiometry therefore seems unlikely. In addition, the mRNA levels for ActR-II and BMPR-II do not differ between ATDC5 and C2C12 cells, and ActR-IIB seems absent in both cell lines (see Fig. 5c). The only other type I receptors except for BMPR-IA and BMPR-IB that activate SMAD1/5/8 (and therefore could potentially be activated via GDF-5 and BMP-2) are ActR-IC (also known as TSR-I), which within the BMP/GDF subgroup is highly specific for BMP-9 and BMP-10 [37], and ActR-I (also known as Alk2). Although the BMP type I receptor Alk2 was shown to be involved in endochondral ossification via regulation of the proliferation and differentiation of chondrocytes [45], Alk2 seems an unlikely candidate to explain the differences in GDF-5 signaling. Alk2 was shown to require N-glycosylation at a conserved site in the ligand for binding and activation [46], which is lacking in GDF-5. Thus, irrespective of the fact that the mRNA levels for ActR-I were highly similar in ATDC5 and C2C12 cells, a cell type-dependent differential participation of Alk2 in a GDF-5-based heteromeric receptor complex seems unlikely.

Other components that potentially modulate signal transduction of TGF-β members derive from the large group of modulator proteins, e.g., noggin, follistatin, and members of the chordin and DAN family. These secreted proteins are present in the extracellular space and alter the activities of the ligands by binding to and blocking their interaction with their cellular receptors, thereby essentially decreasing the concentration of the “free” ligand. The interactions of these modulators can exhibit remarkable ligand specificity even for closely related ligands such as BMP-6 and BMP-7 [47]. Owing to the modulator’s own ligand-specificity profile, these proteins might thus individually fine-tune the active concentration for each TGF-β ligand present at the site of action, thereby establishing a defined equilibrium for a set of ligands that together yield an accurately defined biological response in the target cells. Whereas such a simple ligand-dependent modulatory mechanism would be consistent with the higher concentrations required for GDF-5 to initiate induction of ALP expression in C2C12 cells, it does not fit our observation that GDF-5 can effectively antagonize BMP-2 in these cells. The latter requires that GDF-5 binds to the same receptor assembly through which BMP-2 is signaling without activating this receptor, and thus a decrease in the concentration of free GDF-5 through the actions of a modulator protein is in contrast with its antagonistic property.

This strongly points towards an additional membrane-located component(s), whose presence differs in the different cellular environments and which participates either in the ligand-receptor complex of GDF-5 or of BMP-2, and thereby influences the signaling output of that factor. For instance, the presence of such an additional component in ATDC5 cells (or in the calvaria) could render GDF-5 signaling active in this context. But in C2C12 cells (or at heterotopic sites), where this component is missing, GDF-5 would still assemble a receptor complex composed of the same BMP receptors and thus be able to antagonize BMP-2 activity, but unable to induce—at least parts of—the downstream signaling cascade. One group of molecules that fulfills these criteria comprises the various BMP/TGF-β pseudo-receptors and co-receptors. Some of these co-receptors adjust the signaling potential of particular ligands via the relatively simple mechanism of enhancing the ligand’s binding affinities to the sites of action. For instance, glypicans, which are multifunctional glycosylphosphatidylinisotol (GPI)-anchored heparin sulfate proteoglycans, were found to modulate the signaling cascades of regulatory proteins such as Wnt, Sonic Hedgehog, TGF-β members, and Fibroblast Growth Factors (FGFs) [48]. Six glypican members are found in humans and mice and, for the glypican LON-2 of *Caenorhabditis elegans* as well as the glypican Dally of *Drosophila*, direct binding to BMP members has been shown [49, 50]. Glypican 3 and BMP-4 likely cooperate during limb and skeletal development given that heterozygous mutations in *bmp-4* in combination with hemizygous mutations in *gpc-3* dramatically enhance the polydactyly phenotype seen in *bmp-4* heterozygous mice [51]. Another BMP-specific group of co-receptors is the so-called repulsive guidance molecules (RGMs) co-receptor family, i.e., DRAGON/RGMb, RGMa, and Hemojuvelin/RGMc, which initially were thought to act via an affinity-enhancing mechanism [52]. Owing to their nature as GPI-anchored proteins without an intracellular domain, a direct influence on signaling other than enhancing the local concentration of the ligand seems impossible. However, RGMa was shown to alter type II receptor specificity, possibly leading to a qualitatively different signaling output when RGMa is present [53]. Recent structural/functional analyses of the RGM–BMP interaction have revealed an even more complex picture [54]. Because the binding site of RGM co-receptors on BMPs highly overlaps that for BMP type I receptors, a strong attenuation of BMP signaling would be expected in the presence of RGM co-receptors, the opposite of what is observed [52, 55, 56]. Siebold and colleagues suggested that upon internalization of BMP–RGM-type II receptor complexes by endocytosis, these complexes might be targeted to endosomal compartments. Here, BMP dissociates from the RGM co-receptor owing to the pH-dependent nature of this interaction and forms a ternary complex with a BMP type I and type II receptor, the former of which can undergo constitutive endocytosis [54]. Signaling from this compartment might then have a different output, allowing for ligand-specific and context-specific signaling ([54], see also [57]). Could one of the members of the RGM family therefore represent the sought-after GDF-5 co-receptor? Again, because the expression levels for all three RGM members seem similar in GDF-5-responsive (ATDC5) as well as non-responsive cells (C2C12), it seems very unlikely that their presence or absence alters the cell responsiveness to GDF-5.

The most pronounced effect on ligand-mediated signaling is linked to the presence of the co-receptors betaglycan (TβR-III) or cripto. Betaglycan can act as a co-receptor in TGF-β signaling [58], but is also required for inhibin function [59]. In the presence of betaglycan, inhibin binds the type II receptors ActR-II, ActR-IIB, and BMPR-II, thereby inhibiting not only activin, but also signaling of certain BMPs (i.e., BMP-2, BMP-7, and GDF-5) [60]. A similar modulating function was described for the GPI-anchored membrane protein cripto, which is required for receptor assembly and activation of members of the nodal family [61] and GDF-1/Vg1[62]. Cripto binds nodal independently of TGF-β receptors via its EGF-like domain and ActR-IB via its CFC domain [63, 64], thus facilitating signaling of this ligand. Cripto also binds to activin but only in the presence of ActR-II/IIB. At the same time, cripto binding to activin blocks the interaction with ActR-IB and subsequently prevents the ligand-mediated association of ActR-IB with ActRII/IIB [65]. Thus, cripto controls the differential use of (or access to) the activin receptor by different ligands. These regulatory properties of betaglycan and cripto are reminiscent of our observations obtained from in vitro as well as in vivo experiments in that a not-yet identified co-receptor possibly triggers the biological activity of GDF-5. Theoretically, two opposing mechanisms are feasible: The first mechanism involves an antagonizing GDF-5 specific co-receptor, whose presence still allows binding of GDF-5 to its cognate receptors, but prevents subsequent processes required for certain biological responses such as the induction of ALP gene expression. This hypothetical inhibiting co-receptor would be present in vivo in muscular tissue or—in our cell line model—in C2C12 cells. Given that BMP-2 and GDF-5 utilize the same subset of BMP type I and type II receptors, this hypothetical inhibitor would enable the antagonistic properties of GDF-5. The second mechanism postulates an activating co-receptor specifically required for GDF-5 to signal. In this case, the co-receptor must be present in tissue or cells in which osteogenic markers—like in the calvarial model or in ATDC-5 cells—are inducible upon GDF-5 addition. The latter mechanism seems more likely, because addition of GDF-5 led to SMAD activation in ALP-positive ATDC-5 as well as in ALP-negative C2C12 cells, showing that a defined subset of signals can still be transduced in the absence of the GDF-5-specific activating co-receptor.

## Conclusions

Besides the aspect that GDF-5 cannot induce the full signaling cascade in all cell lines and tissues but possibly requires additional components for this functionality, our study also revealed a remarkable BMP-2 antagonizing function for GDF-5, which is dependent on the cellular context. This is of particular interest because GDF-5 can possibly inhibit the process of ossification mediated by BMP-2 and BMP-4. GDF-6 might share the same function, because Shen *et al*. showed a GDF-6-dependent inhibition of osteogenic differentiation of mesenchymal stem cell [66]. The BMP-2 antagonistic function of GDF-5 and possibly also of GDF-6 might shed new light onto the joint forming process. Possibly GDF-5 and GDF-6 are only required for a fine-tuning of pro-chondrogenic/osteogenic and anti-chondrogenic/osteogenic stimuli caused by other BMPs or modulator proteins such as noggin. Such a scenario would explain the relatively mild phenotypes of *gdf-5* null mice as well as those caused by different activating or deactivating *gdf-5* mutations in human. A fascinating aspect in this context is that malformations of particular joints are coherently linked to certain mutations in GDF-5. It can therefore be assumed that every joint requires an individually fine-balanced equilibrium of pro-chondrogenic and anti-chondrogenic factors.

## Methods

### Expression and purification of recombinant proteins

Expression and purification of *Escherichia coli*-derived recombinant human BMP-2, human GDF-5, and the mutant GDF-5 R57A was carried out as previously described [19, 26, 67]. The extracellular domain of mature human BMPR-IA [Swiss-Prot:P36894] comprising residues 24–152 was expressed in *E. coli* as thioredoxin-fusion protein using a protocol similar to those previously described [25, 26, 68]. Purity and homogeneity of the recombinant proteins were validated by sodium dodecyl sulfate polyacrylamide gel electrophoresis (SDS-PAGE), analytical reverse-phase high-performance liquid chromatography and electrospray ionization Fourier transform ion cyclotron resonance mass spectrometry. For additional quality control, the affinities for binding of the BMP-2 and GDF-5 proteins to BMPR-IA and other type I and type II receptors were determined by surface plasmon resonance as previously described and compared to published data [17].

### Crystallization of the binary complex GDF-5 R57A:BMPR-IA

The binary complex of the GDF-5 variant R57A bound to the extracellular domain of BMPR-IA was obtained and purified similar to the complex GDF-5:BMPR-IB_EC_ [68]. Full details of the preparation of the complex and the crystallization have been published elsewhere [67]. Crystals of different morphology were grown from vapor diffusion setups using magnesium formate, ammonium citrate, or polyethylenglycols of various length as precipitants and with pH ranging from 5.5 to 8.0. Crystallization conditions were optimized by varying temperature, buffer chemistry, and pH, as well as the concentration of the precipitants. Only crystals exhibiting a hexahedral or rhombohedral form diffracted X-rays to high resolution and were thus suitable for data acquisition. Those crystals with final dimensions of 300 × 200 × 80 μm were obtained within 4 weeks from 0.1 M HEPES pH 7.5, 0.2 M NaCl, and 20 % PEG 3350 at room temperature.

### Data acquisition, processing, and structure analysis

The high-resolution native data were collected from a single crystal at 100 K using a home source (MicroMax007 Xray generator, VariMax HighRes optics and a R-AXIS IV++ image plate system, Rigaku, The Woodlands, Texas, USA). Diffraction data with a maximal resolution of 2.28 Å were obtained from a 123 ° sweep using 0.5 ° oscillation and 360 s exposure time per frame. The data were indexed and scaled using the software CrystalClear 1.3.6 (Rigaku). The crystal of the GDF-5 R57A:BMPR-IA_EC_ complex belongs to the spacegroup I_2_ with unit cell constants of a = 63.81 Å, b = 62.85 Å, c = 124.99 Å, and β = 95.85 °. Initial phasing was performed by molecular replacement using the software package CNS 1.2 and the structure of the binary complex of BMP-2 bound to BMPR-IA [PDB:1REW] as a search template; full technical details of the initial phasing analysis have been published elsewhere [67]. The amino acid sequence of the BMP-2 template was adapted by manual rebuilding to cover the sequence of GDF-5 R57A using the software Quanta2008 (MSI Accelrys, San Diego, CA, USA). The structure of the binary complex GDF-5 R57A:BMPR-IA was then refined by iterative cycles of manual rebuilding using Quanta2008 and refinement using Refmac version 5.02 of the software package CCP4. During refinement, one TLS group was defined per molecule chain (one TLS group per GDF-5 monomer and one TLS group for each BMPR-IA_EC_ molecule) to account for anisotropy in the data. The progress of refinement was monitored by cross-validation using a test data set comprising 5 % of the reflections. In the final refinement cycles F_obs_-F_calc_ difference electron density maps were used to identify water molecules located within 5 Å of the protein complex. The final structure of the GDF-5 R57A:BMPR-IA_EC_ complex comprised 3,015 protein atoms and 93 water molecules. The R-factor was 0.19 for R_cryst_ and 0.24 for R_free_. Statistics for data processing, refinement, and structure quality are given in Table 2. The structure has been deposited at the RCSB databank [PDB:3QB4] [69].

**Table 2:** Data collection and refinement statistics for the GDF-5 R57A:BMPR-IA complex structure

Processing	
Space group	I2
Unit cell	a = 63.81 Å, b = 62.85 Å, c = 124.99 Å α = γ = 90 °, β = 95.85 °
Wavelength (Å)	1.5418
Resolution (Å)	24.0–2.28 (2.40–2.28)
No. reflections	51,824 (7,550)
No. unique reflections	22,044 (3,177)
R_merge_	0.066 (0.384)
<(I)/σ (I)>	7.4 (2.0)
Completeness (%)	97.8 (97.4)
Redundancy	2.4 (2.4)
Refinement	
Resolution (Å)	23.8–2.28 (2.34–2.28)
*R*_work_/*R*_free_ (%)	19.7 (25.8)/24.1 (34.4)
Correlation coefficient (F_obs_-F_calc_)_free_	0.938
Coordinate error (ESU) based R_free_	0.218 Å
Number atoms	
Protein	3,015
Water	93
R.m.s. deviations	
Bond length (Å)	0.013
Bond angles (°)	1.457
Torsion angles (°)^b^	8.345
Torsion angles (°)^c^	19.839
TLS	4 groups GDF-5 R57A (chain A + C, residues 11–115, 10–115) BMPR-IA (chain B + D, residues 31–118, 31–119)
Ramachandran analysis^d^	
Number of residues in favored region	363 (95.5 %)
Number of residues in allowed region	16 (4.2 %)
Number of residues in outlier region	1 (0.3 %)

Values in parentheses are for the highest-resolution shell

^a^One crystal was used to collect the diffraction data

### Alkaline phosphatase assay

The teratocarcinoma AT508-derived cell line ATDC-5 (RIKEN, No. RCB0565) was cultured in a 1:1 ratio of Dulbecco’s modified eagle medium (DMEM) and Ham’s F12 medium containing 5 % (v/v) fetal calf serum (FCS), and antibiotics (100 units ml^−1^ of penicillin G and 100 μg ml^−1^ of streptomycin). Promyoblastic C2C12 cells (ATCC CRL-172) were grown in DMEM containing 10 % (v/v) FCS and the same antibiotics. For ALP assays, the cells were serum-starved (2 % FCS) and exposed to ligands or to mixtures of ligands and neutralizing antibodies for 72 h in 96-well microplates. After cell lysis, ALP activity was measured by *p*-nitrophenylphosphate conversion using an enzyme-linked immunosorbent assay reader at 405 nm. All experiments were performed two times in duplicate. ALP assays using the neutralizing BMPR-IA antibody were performed in two independent experiments. For data presentation of raw data (non-normalized), a single experiment is shown in Fig. 6a, the duplicate is shown in Additional file 6: Figure S5.

### Quantification of BMP-receptors and co-receptors by qRT-PCR

RNA of C2C12 or ADTC-5 cells was extracted using the RNeasy Plus Mini Kit (Qiagen, Hilden, Germany) according to the supplier’s instructions. Purified RNA was reversely transcribed by murine Moloney leukemia virus reverse transcriptase using random hexamer oligonucleotides. cDNA was subjected to qRT-PCR. The experiments were performed in duplicate. The data were normalized to *HPRT* expression (for primer sequences and statistics, see Additional file 4: Table S1).

### Western blot analysis

To determine of SMAD or p38 MAP kinase activity cells were simulated with either 10 nM of BMP-2, 10 nM of GDF-5 R57A or 100 nM of GDF-5 for the indicated time points. Cells were lysed and cellular debris was subsequently removed by centrifugation. Protein-containing supernatants were separated by SDS-PAGE and blotted onto nitro-cellulose membranes. The membranes were blocked and incubated with the indicated antibodies (anti-SMAD-1, -p-SMAD-1, -p38 MAPK, and -P-p38 MAPK; Cell Signaling Technology, Danvers, MA, USA). For detection, secondary antibodies coupled to horseradish peroxidase were used (anti-mouse IgG, anti-rabbit IgG; Cell Signaling Technology, Danvers, MA, USA). Chemoluminescence was analyzed using a Fluor Chem Q gel documentation system (Cell Biosciences, Victoria, Australia).

### Preparation of carrier derived from equine bone

Equine bone was freshly harvested and cleaned from the surrounding soft tissues. Blocks of cancellous bone were cut and stored at −80 °C until further processing. After thawing, the bone marrow and fat was removed by centrifugation at 1000 × g. The cancellous blocks were then cleaned with pressurized steam. After defatting in methanol and chloroform (1:1) the bone was washed in distilled water and cut under constant irrigation into slices of 10 or 1 mm thickness. The slices were then demineralized in 0.5 M HCl. Completion of demineralization was assessed by radiographic analysis. For calvarial reconstruction, discs of 8 mm in diameter and 1 mm height were punched out of the thin soft bone matrix. For heterotopic implantation, cylinders of 5 mm in diameter and 10 mm thickness were cut out of the thick matrix sheets. All carriers were sterilized in a methanol and chloroform mix (1:1) and washed in sterile deionized water. For storage, implants were lyophilized and kept at room temperature.

For doping with growth factors (4, 20, or 80 µg; 4 µg of BMP-2 serve as reference), the lyophilized matrix was hydrated in sterile deionized water. Growth factors were then applied in 40 μl of aqueous solution evenly spread on the porous carrier. Finally, the doped carriers were lyophilized and stored at 4 °C in an air-sealed sterile container.

### Surgery

Male adult Sprague Dawley rats with a body weight of 400 ± 50 g (Charles River Breeding Laboratories, Sulzfeld, Germany) were used for all studies. They were caged in groups of four and fed ad libitum with a standard laboratory pelleted diet and water. Animals and wound conditions were monitored daily. For surgery, a mixture of ketamine hydrochloride (Ketavet (Parke-Davis, Berlin, Germany); 120 mg/kg weight) and Xylazine (Rompun (Bayer, Wuppertal, Germany); 5 mg/kg weight) was intraperitoneally administered for anesthesia. Circulatory parameters were monitored using a pulse oximeter attached to the tail root. Heterotopic implantation was performed by shaving the hind legs and subsequent disinfection with 70 % ethanol. Following a small skin incision a muscular pouch was formed by blunt preparation. The cylindrical implants were inserted and positioned parallel to the diaphysis of the femur. The pouch as well as the skin was closed with a resorbing suture.

Orthotopic implantation in calvarial defects was performed after disinfection, transverse skin incision, and resection of the periosteum in the area of craniotomy. The craniotomy defect of 8 mm in diameter was prepared with a trephine in a slow-speed dental hand piece under constant irrigation with sterile 0.9 % physiologic saline. The calvarial disk was dissected free, avoiding dural perforations and keeping the sagittal venous sinus intact. The lyophilized implant was directly placed in the defect and held in place until it was fully soaked with blood. The swelling following the rehydration of the matrix ensured a tight and secure fit of the implanted discs. The skin was closed with a resorbing suture.

### Quantitative radiograph analysis

The animals were killed 28 days after implantation and the specimens were retrieved, including the soft tissue surrounding the calvaria bone. Radiographs of the specimen were taken using a FAXITRON X-Ray unit model 44385 (Hewlett Packard, McMinnville, OR, USA). Heterotopic implants were radiographed with 22 kV for 27 s. The explanted calvaria were placed with the external cortical layer facing the film (Kodak Industrex SR*)* and exposed with 20 kV for 30 s. The radiographs were scanned with a Nikon Coolscan LS 2000 slide scanner at 600 dpi as 256-bit gray-scale images. Image analysis was performed using the program NIH Image 1.61 (developed at the US National Institutes of Health and available on the Internet at [70]) in a Windows version (Scion Corporation). Bone regeneration was assessed by measuring the area of newly formed bone in the defect site. Briefly, the median- and mean cortical densities and their standard deviations (M_cd,_ SD_cd_) were determined over a wide range of non-operated calvarial bone. The thresholds of gray levels were set to M_cd_ ± 2*SD_cd_ to mark the total bone. The sites that were initially defective were outlined manually and the area of newly formed bone was measured from the latter images. For statistics see Additional file 8: Tables S2 and Additional file 9: Table S3.

### Ethics approval

All animal experiments were approved by the local ethical committee and by the veterinary authorities (Regierung von Unterfranken, D-97070 Würzburg, No. 55.2-2531.01-80/07).

### Availability of supporting data

The structure has been deposited at RCSB databank [PDB:3QB4].

## Additional files

Additional file 1: Figure S1. Overview of the symmetrical architecture of the GDF-5-R57A:BMPR-IA complex. (TIFF 3812 kb) Additional file 2: Figure S2. Orientation of BMPR-IA_EC_ in complex with different ligands. (TIFF 2535 kb) Additional file 3: Figure S3. Comparison of ALP-inducing potentials of BMP-2 and the GDF-5 variants in ATDC-5 cells. (JPEG 2403 kb) Additional file 4: Table S1. Raw data from qRT-PCR experiments (Fig. 6c, d). (DOCX 20 kb) Additional file 5: Figure S4. GDF-5-mediated induction of ALP expression in transfected ATDC-5 cells. (JPEG 2452 kb) Additional file 6: Figure S5. Inhibition of ligand-induced signal transduction (biological replicate of the experiment shown in Fig. 6a). (JPEG 2710 kb) Additional file 7: Figure S6. Time-dependent ligand-induced Smad and p38 mitogen activated protein (MAP)-kinase activation. (JPEG 3079 kb) Additional file 8: Table S2. Statistical data from in vivo experiments (calvarial defect/orthotopic model). (DOCX 15 kb) Additional file 9: Table S3. Statistical data from in vivo experiments (hind limb/heterotopic model). (DOCX 16 kb)

## Abbreviations

ALP: alkaline phosphatase
BMP: bone morphogenetic protein
BMPR: BMP receptor
DMEM: Dulbecco’s modified eagle medium
EC: extracellular domain
EMP: EPO mimetics
EPO: erythropoietin
FCS: fetal calf serum
GDF: growth and differentiation factor
GPI: glycosylphosphatidylinisotol
hGH: human growth hormone
qRT-PCR: quantitative reverse transcription polymerase chain reaction
RGM: repulsive guidance molecules
SDS-PAGE: sodium dodecyl sulfate polyacrylamide gel electrophoresis
TGF-β: transforming growth factor β
