# Supplementary figures and images for: GDF-5 can act as a context-dependent BMP-2 antagonist
Source: BMC Biol. 2015 Sep 18;13:77. doi: 10.1186/s12915-015-0183-8 (PMC4575486; doi:10.1186/s12915-015-0183-8)

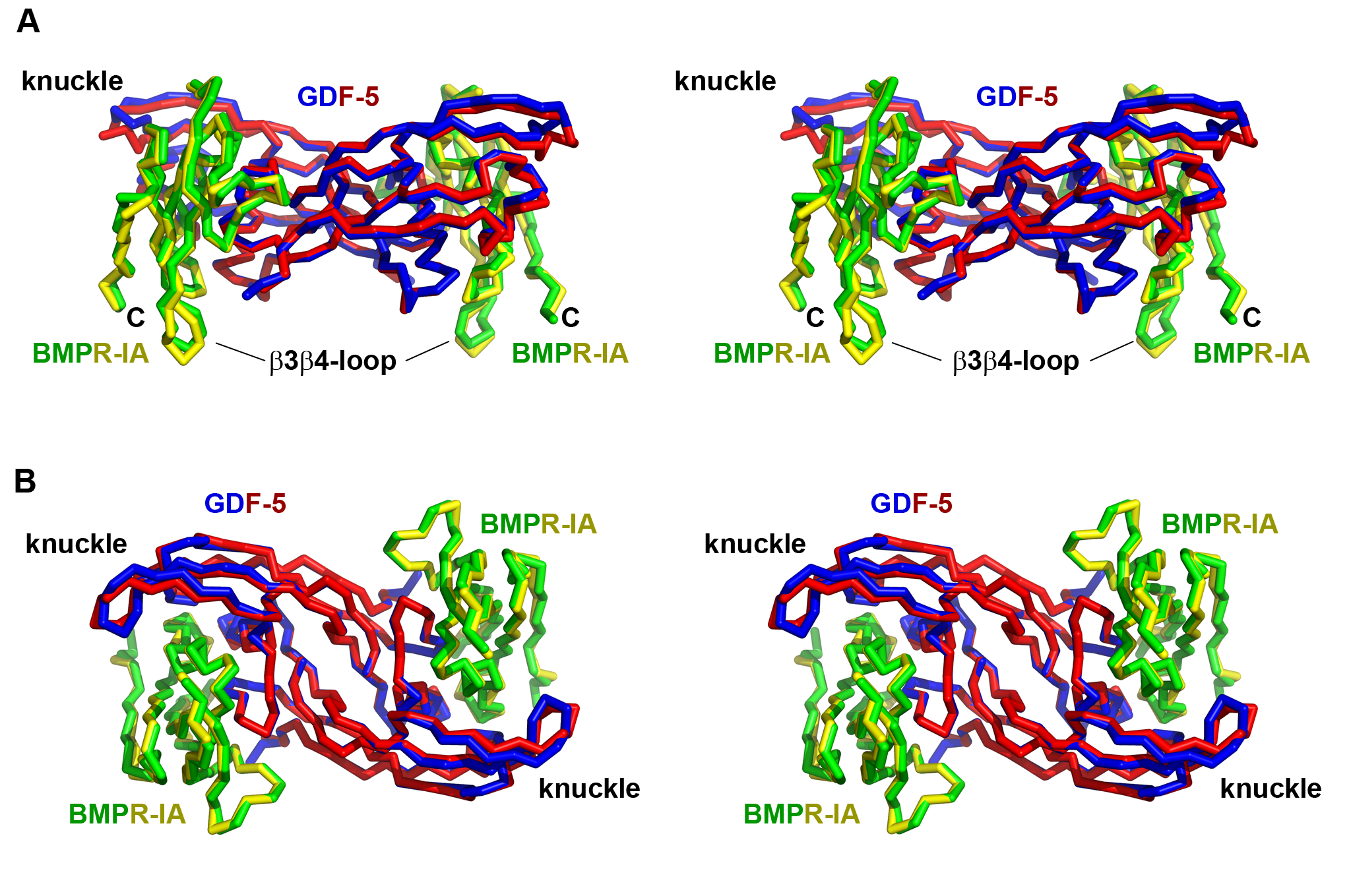

Supplement: Additional file 1: Figure S1. — Overview of the symmetrical architecture of the GDF-5-R57A:BMPR-IA complex. (TIFF 3812 kb) [file 12915_2015_183_MOESM1_ESM.tif]

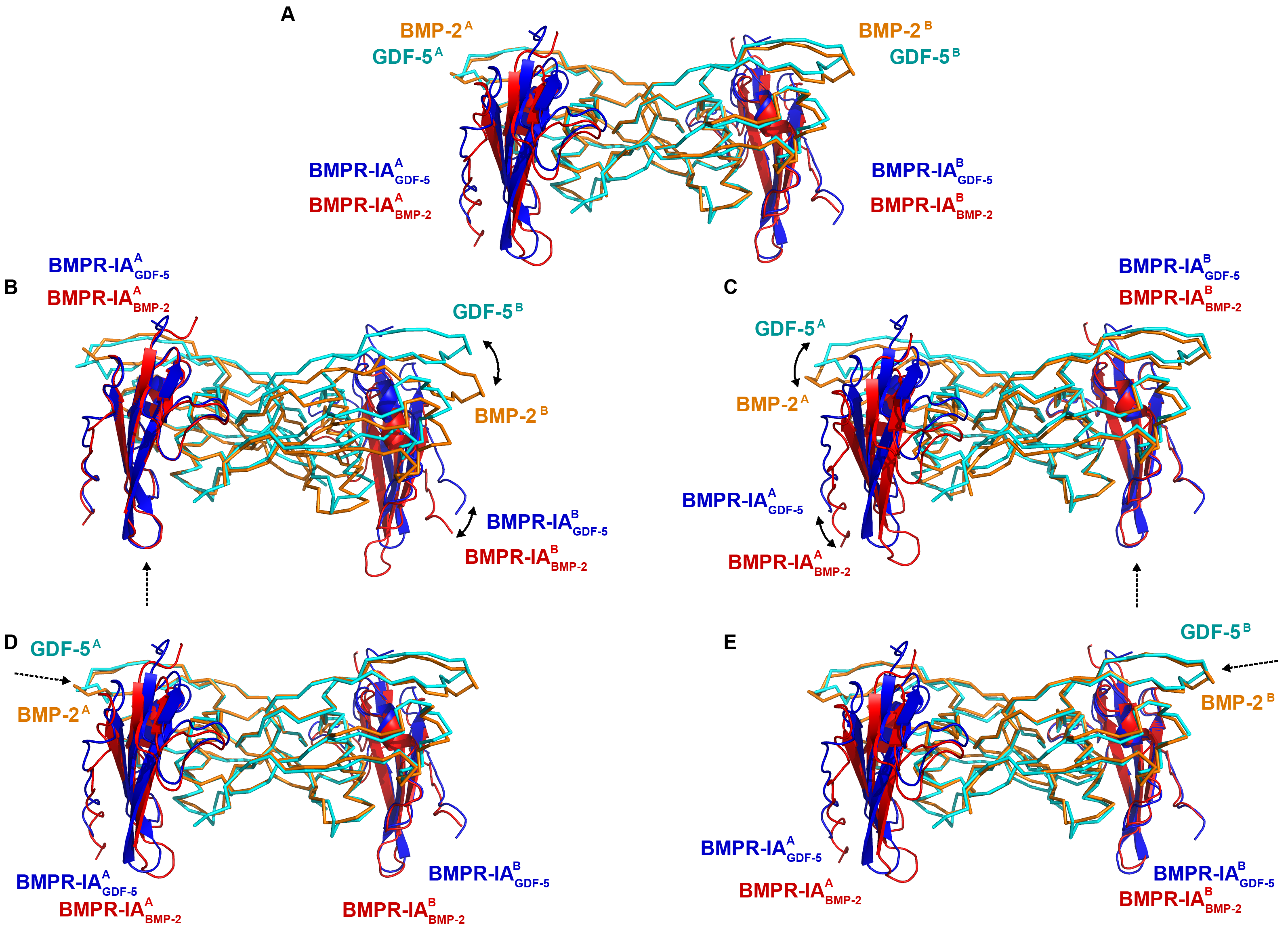

Supplement: Additional file 2: Figure S2. — Orientation of BMPR-IAEC in complex with different ligands. (TIFF 2535 kb) [file 12915_2015_183_MOESM2_ESM.tif]

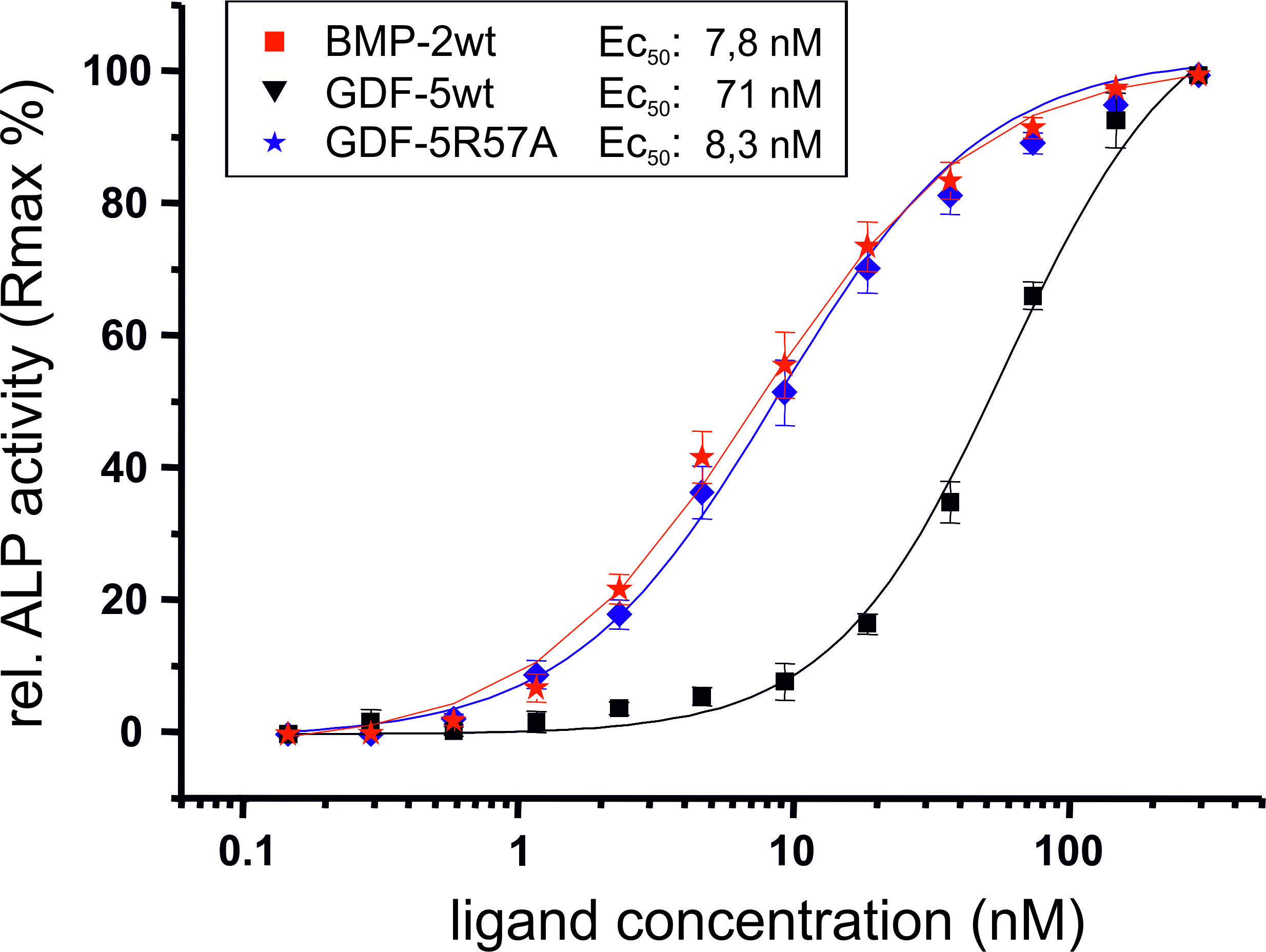

Supplement: Additional file 3: Figure S3. — Comparison of ALP-inducing potentials of BMP-2 and the GDF-5 variants in ATDC-5 cells. (JPEG 2403 kb) [file 12915_2015_183_MOESM3_ESM.jpg]

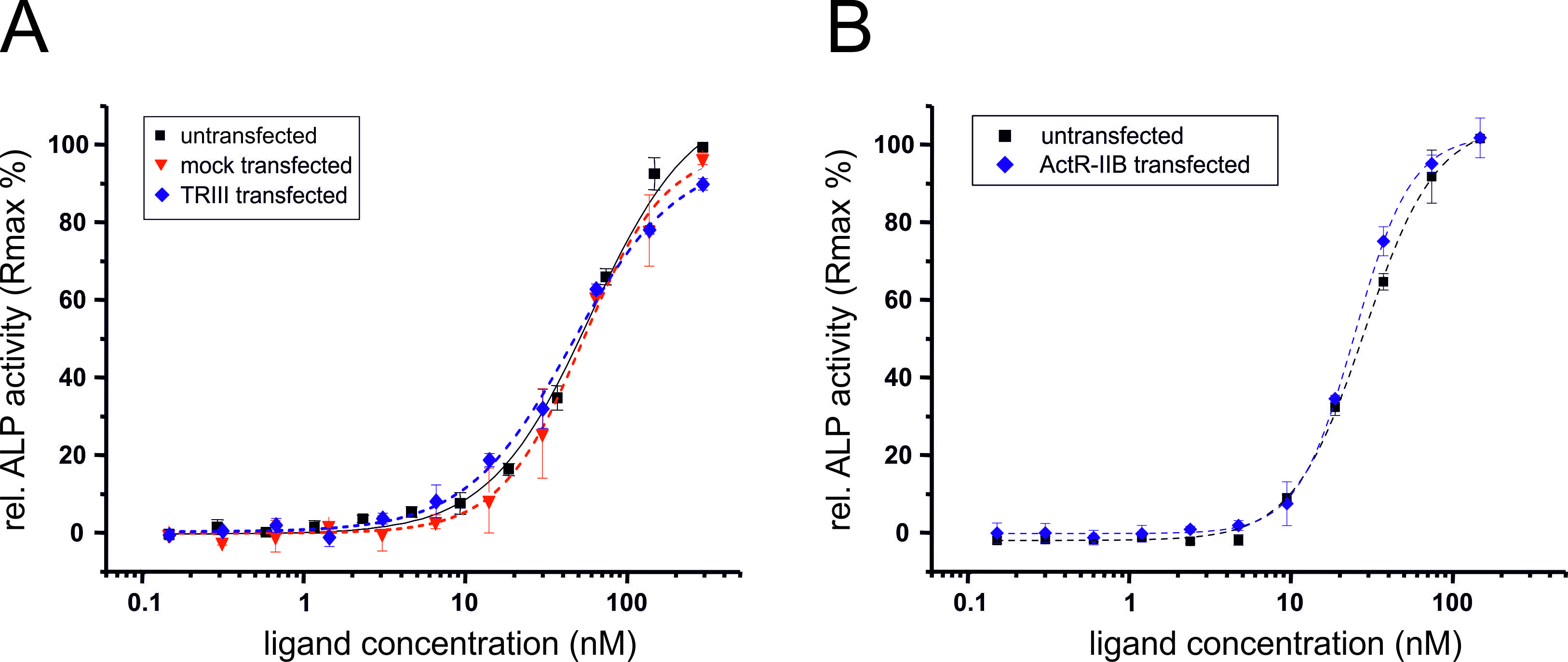

Supplement: Additional file 5: Figure S4. — GDF-5-mediated induction of ALP expression in transfected ATDC-5 cells. (JPEG 2452 kb) [file 12915_2015_183_MOESM5_ESM.jpg]

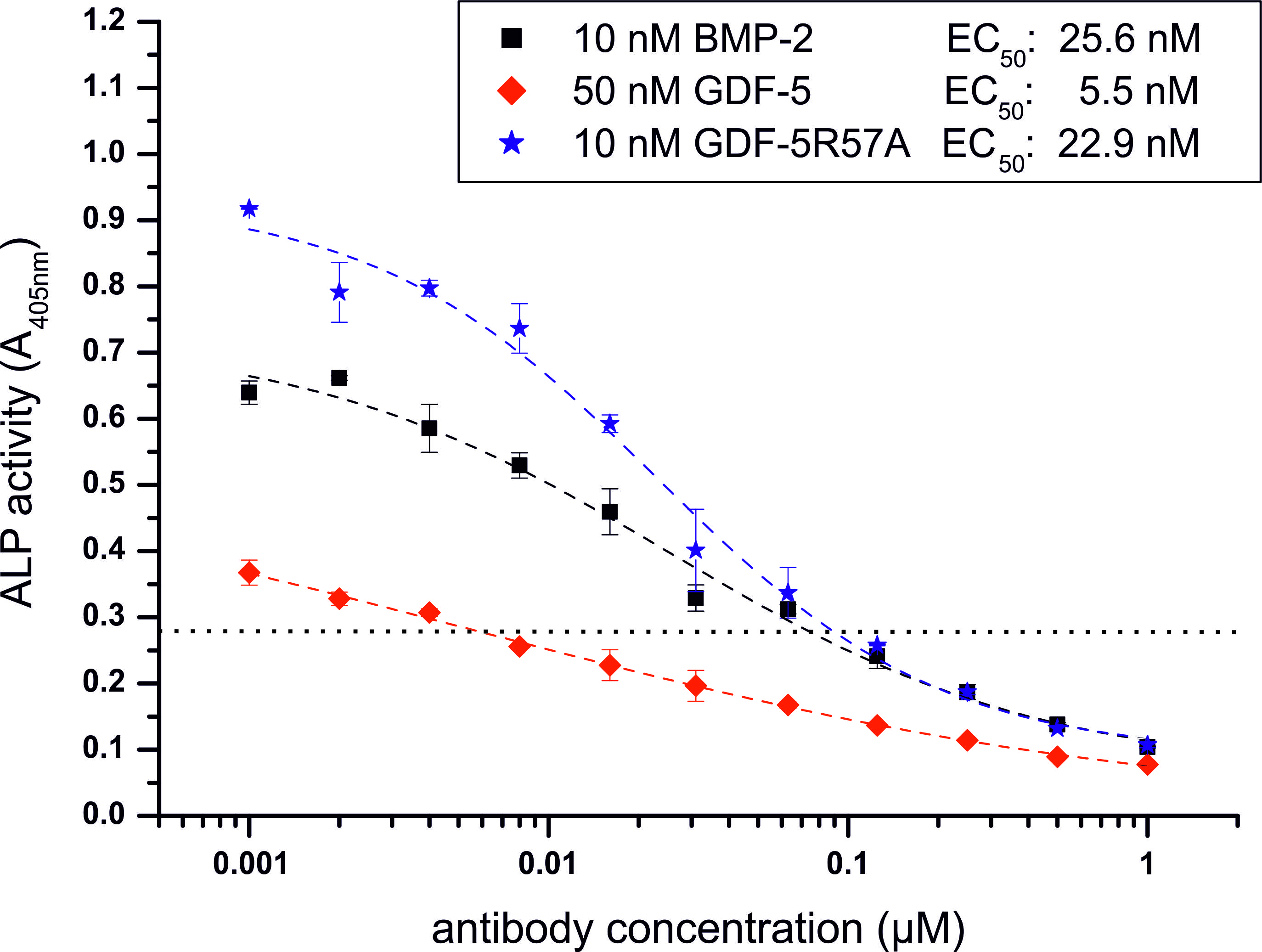

Supplement: Additional file 6: Figure S5. — Inhibition of ligand-induced signal transduction (biological replicate of the experiment shown in Fig. 6a). (JPEG 2710 kb) [file 12915_2015_183_MOESM6_ESM.jpg]

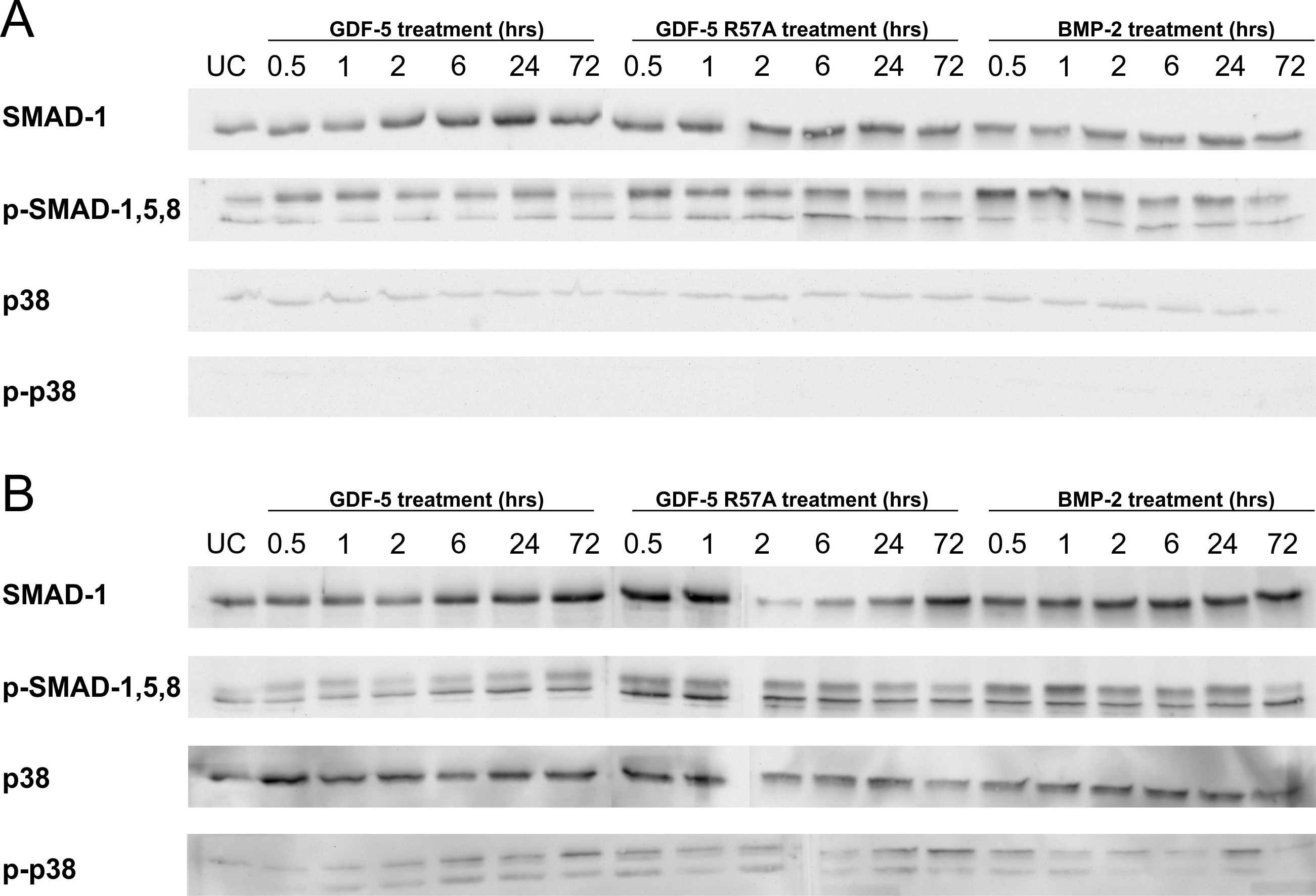

Supplement: Additional file 7: Figure S6. — Time-dependent ligand-induced Smad and p38 mitogen activated protein (MAP)-kinase activation. (JPEG 3079 kb) [file 12915_2015_183_MOESM7_ESM.jpg]
