# Supplementary material for: GDF-5 can act as a context-dependent BMP-2 antagonist
Source: BMC Biol. 2015 Sep 18;13:77. doi: 10.1186/s12915-015-0183-8 (PMC4575486; doi:10.1186/s12915-015-0183-8)
Supplement: Additional file 4: Table S1. — Raw data from qRT-PCR experiments (Fig. 6c, d). (DOCX 20 kb) [file 12915_2015_183_MOESM4_ESM.docx]

| **Table S1** |  |  |  |  |  |  |  |  |
| --- | --- | --- | --- | --- | --- | --- | --- | --- |
|  |  |  |  |  |  |  |  |  |
| Raw-data from qRT-PCR experiments (Figure 6C, 6D) | | | | | |  |  |  |
| n=4; (2 technical-, 2 biological replicates) | | | | |  |  |  |  |
|  |  |  |  |  |  |  |  |  |
|  |  | C2C12 | | ATDC5 | |  | C2C12 | ATDC5 |
|  |  |  |  |  |  |  |  |  |
| 1. Receptors |  | mean | STDev | mean | STDev |  | Fold Expression | Fold Expression |
|  |  | (cycle number) | | (cycle number) | |  | HPRT = 1 | HPRT = 1 |
| prHPRT |  | 20.84 | 0.28 | 21.15 | 0.35 |  | 1.00E+00 | 1.00E+00 |
| BMPR-IA |  | 17.81 | 0.19 | 17.32 | 0.38 |  | 8.10E+00 | 1.44E+01 |
| BMPR-IB |  | 22.33 | 0.25 | 21.25 | 0.56 |  | 3.54E-01 | 6.59E-01 |
| ActR-I |  | 19.56 | 0.31 | 19.91 | 0.42 |  | 2.46E+00 | 2.38E+00 |
| ActR-II |  | 19.79 | 0.42 | 20.88 | 0.71 |  | 1.98E+00 | 1.49E+00 |
| ActR-IIB |  | 26.96 | 0.73 | 33.04 | 0.85 |  | 1.36E-02 | 2.71E-04 |
| BMPR-II |  | 19.13 | 0.13 | 18.67 | 0.19 |  | 3.25E+00 | 5.46E+00 |
| TβR-I |  | 20.14 | 0.19 | 20.44 | 0.35 |  | 1.52E+00 | 1.62E+00 |
| TβR-II |  | 20.30 | 0.28 | 20.55 | 0.64 |  | 1.41E+00 | 1.52E+00 |
|  |  |  |  |  |  |  |  |  |
| 2. Co-Receptors |  | mean | STDev | mean | STDev |  | Fold Expression | Fold Expression |
|  |  | (cycle number) | | (cycle number) | |  | HPRT = 1 | HPRT = 1 |
| TβR-III |  | 21.42 | 0.14 | 28.42 | 0.57 |  | 6.60E-01 | 6.57E-03 |
| Cripto |  | 33.23 | 0.42 | 36.78 | 0.18 |  | 1.85E-04 | 1.81E-05 |
| RGMa |  | 25.96 | 0.64 | 24.71 | 0.35 |  | 2.82E-02 | 8.25E-02 |
| RGMb |  | 21.31 | 0.57 | 22.41 | 0.14 |  | 7.07E-01 | 4.20E-01 |
| RGMc |  | 27.95 | 0.21 | 27.66 | 0.49 |  | 7.04E-03 | 1.10E-02 |
| BAMBI |  | 27.08 | 0.35 | 29.41 | 0.63 |  | 1.31E-02 | 3.28E-03 |
| Endoglin |  | 26.16 | 0.78 | 28.35 | 0.21 |  | 2.45E-02 | 1.54E-01 |
| ROR-2 |  | 24.11 | 0.14 | 23.87 | 0.21 |  | 1.02E-01 | 6.80E-03 |

**Primers used for qRT-PCR**

1. Receptors

ActR-I _sense_ 5´-TGGAAGATGAGAAGCCCAAG-3´

ActR-I _antisense_ 5´-CCTTTAGTGGGCAGCTGGGC-3´

BMPR-IA _sense_ 5´-AGCCCTACATCATGGCTGAC-3´

BMPR-IA _antisense_ 5´-AGCGTTTCACACACACAACC-3´

BMPR-IB _sense_ 5´-GTGCCCAGTGACCCTTCTTA-3´

BMPR-IB _antisense_ 5´-TGACATTTTGGCAAGGGTTT-3´

TβR-I _sense_ 5´-GGTCATGGGAGTGTTCTGGT-3´

TβR-I _antisense_ 5´-TGTGCTGACAGTCCTCTGCT-3´

ActR-II _sense_ 5´-AGGAAGAAATTGGCCAGCAT-3´

ActR-II _antisense_ 5´-CCAGCTGATAACCTGGCTTC-3´

ActR-IIB _sense_ 5´-ACTTCGGCCTGGCTGTTCGG-3´

ActR-IIB _antisense_ 5´-GAAGGAGCCATCAACTTCCA-3´

BMPR-II _sense_  5´-TGTATGCGCTTGGACTCATC-3´

BMPR-II _antisense_ 5´-TCTGGGAACTTGGGTCTCTG-3´

TβR-II _sense_ 5´-CCATGGCTCTGGTACTCTGG-3´

TβR-II _antisense_ 5´-CGGTCAAAGTCTCACACACG-3´

HPRT _sense_ 5´-AGTCAACGGGGGACATAAAA-3´

HPRT _antisense_ 5´-AGAGGTCCTTTTCACCAGCA-3´

2. Co-Receptors

The primers for co-receptors were purchased from Qiagen (Hilden, Germany).
