# Supplementary material for: GDF-5 can act as a context-dependent BMP-2 antagonist
Source: BMC Biol. 2015 Sep 18;13:77. doi: 10.1186/s12915-015-0183-8 (PMC4575486; doi:10.1186/s12915-015-0183-8)
Supplement: Additional file 8: Table S2. — Statistical data from in vivo experiments (calvarial defect/orthotopic model). (DOCX 15 kb) [file 12915_2015_183_MOESM8_ESM.docx]

**Table S2**

Ligand induced bone formation *in vivo.* Calvarial defect (Orthotopic) model.

| **Group (n=6)** | **median [mm²]** | **mean [mm²]** | **SD [mm²]** |
| --- | --- | --- | --- |
| 4 µg wt BMP-2 | 40,65 | 39,68 | 7,77 |
|  |  |  |  |
| 0.4 µg GDF-5 R57A | 16,61 | 17,20 | 5,22 |
| 4 µg GDF-5 R57A | 29,90 | 31,99 | 7,69 |
| 40 µg GDF-5 R57A | 45,98 | 41,13 | 10,19 |
| 80 µg GDF-5 R57A | 47,25 | 44,35 | 5,07 |
|  |  |  |  |
| 4 µg wt GDF-5 | 8,52 | 12,13 | 6,89 |
| 40 µg wt GDF-5 | 21,55 | 24,60 | 5,75 |
| 80 µg wt GDF-5 | 34,28 | 38,75 | 8,75 |

The data represent median and mean values and the respective standard deviations of ossified areas after scaffold explantation.
