# Supplementary material for: GDF-5 can act as a context-dependent BMP-2 antagonist
Source: BMC Biol. 2015 Sep 18;13:77. doi: 10.1186/s12915-015-0183-8 (PMC4575486; doi:10.1186/s12915-015-0183-8)
Supplement: Additional file 9: Table S3. — Statistical data from in vivo experiments (hind limb/heterotopic model). (DOCX 16 kb) [file 12915_2015_183_MOESM9_ESM.docx]

**Table S3**

Ligand induced bone formation in vivo (heterotopic model)

| **Group (n=6)** | **median [mm^2^]** | **mean [mm²]** | **SD [mm²]** |
| --- | --- | --- | --- |
| 4 µg wtBMP-2 | 40.60 | 39.70 | 8.15 |
|  |  |  |  |
| 4 µg GDF-5 R57A | 11.22 | 7.59 | 6.36 |
| 40 µg GDF-5 R57A | 24.95 | 28.50 | 9.02 |
| 80 µg GDF-5 R57A | 48.23 | 46.04 | 4.46 |
|  |  |  |  |
| 4 µg wtGDF-5 | 0.00 | 0.00 | 0.00 |
| 40 µg wtGDF-5 | 0.00 | 0.00 | 0.00 |
| 80 µg wtGDF-5 | 0.00 | 0.00 | 0.00 |
|  |  |  |  |
| 4 µg GDF-5 R57A + 4 µg wtBMP-2 | 10.98 | 9.14 | 8.37 |
| 40 µg GDF-5 R57A + 4 µg wtBMP-2 | 19.94 | 16.37 | 11.99 |
| 80 µg GDF-5 R57A + 4 µg wtBMP-2 | 46.40 | 47.67 | 6.79 |
|  |  |  |  |
| 4 µg wtGDF-5 + 4 µg wtBMP-2 | 26.27 | 27.51 | 3.06 |
| 40 µg wtGDF-5 + 4 µg wtBMP-2 | 17.51 | 19.32 | 5.10 |
| 80 µg wtGDF-5 + 4 µg wtBMP-2 | 4.61 | 3.41 | 2.39 |

The data represent median and mean values and the respective standard deviations of ossified areas after scaffold explantation.
